# Early warning systems for identifying severe maternal outcomes: findings from the WHO global maternal sepsis study

**DOI:** 10.1016/j.eclinm.2024.102981

**Published:** 2024-12-06

**Authors:** Yamikani Chimwaza, Alexandra Hunt, Livia Oliveira-Ciabati, Laura Bonnett, Edgardo Abalos, Cristina Cuesta, João Paulo Souza, Mercedes Bonet, Vanessa Brizuela, David Lissauer, World Health Organization Global Maternal Sepsis Study Research Group, Yamikani Chimwaza, Alexandra Hunt, Livia Oliveira-Ciabati, Laura Bonnett, Edgardo Abalos, Cristina Cuesta, João Paulo Souza, Mercedes Bonet, Vanessa Brizuela, David Lissauer, Mohammad Iqbal Aman, Bashir Noormal, Marisa Espinoza, Julia Pasquale, Charlotte Leroy, Kristien Roelens, Griet Vandenberghe, M. Christian Urlyss Agossou, Sourou Goufodji Keke, Christiane Tshabu Aguemon, Patricia Soledad Apaza Peralta, Víctor Conde Altamirano, Rosalinda Hernández Muñoz, José Guilherme Cecatti, Carolina Ribeiro do Valle, Vincent Batiene, Kadari Cisse, Henri Gautier Ouedraogo, Kannitha Cheang, Phirun Lam, Tung Rathavy, Elie Simo, Pierre-Marie Tebeu, Emah Irene Yakana, Javier Carvajal, María Fernanda Escobar, Paula Fernández, Lotte Berdiin Colmorn, Jens Langhoff-Roos, Wilson Mereci, Paola Vélez, Yasser Salah Eldin, Alaa Sultan, Alula M. Teklu, Dawit Worku, Richard Adanu, Philip Govule, Charles Noora Lwanga, William Enrique Arriaga Romero, María Guadalupe Flores Aceituno, Carolina Bustillo, Bredy Lara, Vijay Kumar, Vanita Suri, Sonia Trikha, Irene Cetin, Serena Donati, Carlo Personeni, Guldana Baimussanova, Saule Kabylova, Balgyn Sagyndykova, George Gwako, Alfred Osoti, Zahida Qureshi, Raisa Asylbasheva, Aigul Boobekova, Damira Seksenbaeva, Saad Eddine Itani, Meilė Minkauskienė, Diana Ramašauskaitė, Owen Chikhwaza, Luis Gadama, Eddie Malunga, Haoua Dembele, Hamadoun Sangho, Fanta Eliane Zerbo, Filiberto Dávila Serapio, Nazarea Herrera Maldonado, Juan I. Islas Castañeda, Tatiana Cauaus, Ala Curteanu, Victor Petrov, Yadamsuren Buyanjargal, Seded Khishgee, Bat-Erdene Lkhagvasuren, Amina Essolbi, Rachid Moulki, Zara Jaze, Arlete Mariano, Nafissa Bique Osman, Hla Mya Thway Einda, Thae Maung Maung, Khaing Nwe Tin, Tara Gurung, Amir Babu Shrestha, Sangeeta Shrestha, Kitty Bloemenkamp, Marcus J. Rijken, Thomas Van Den Akker, María Esther Estrada, Néstor J. Pavón Gómez, Olubukola Adesina, Chris Aimakhu, Bukola Fawole, Rizwana Chaudhri, Saima Hamid, M. Adnan Khan, María del Pilar Huatuco Hernández, Nelly M. Zavaleta Pimentel, Maria Lu Andal, Zenaida Dy Recidoro, Carolina Paula Martin, Mihaela Budianu, Lucian Pușcașiu, Léopold Diouf, Dembo Guirassy, Philippe Marc Moreira, Miroslav Borovsky, Ladislav Kovac, Alexandra Kristufkova, Sylvia Cebekhulu, Laura Cornelissen, Priya Soma-Pillay, Vicenç Cararach, Marta López, María José Vidal Benedé, Hemali Jayakody, Kapila Jayaratne, Dhammica Rowel, Wisal Nabag, Sara Omer, Victoria Tsoy, Urunbish Uzakova, Dilrabo Yunusova, Thitiporn Siriwachirachai, Thumwadee Tangsiriwatthana, Catherine Dunlop, Marian Knight, Jhon Roman, Gerardo Vitureira, Dinh Anh Tuan, Luong Ngoc Truong, Nghiem Thi Xuan Hanh, Mugove Madziyire, Thulani Magwali, Stephen Munjanja, Adama Baguiya, Mónica Chamillard, Bukola Fawole, Marian Knight, Seni Kouanda, Pisake Lumbiganon, Ashraf Nabhan, Ruta Nadisauskiene, Linda Bartlett, Fernando Bellissimo-Rodrigues, Shevin T. Jacob, Sadia Shakoor, Khalid Yunis, Liana Campodónico, Hugo Gamerro, Daniel Giordano, Fernando Althabe, A. Metin Gülmezoglu

**Affiliations:** a Malawi-Liverpool Wellcome Programme, Blantyre, Malawi; b Department of Women’s and Children’s Health, Institute of Life Course and Medical Sciences, University of Liverpool, Liverpool, United Kingdom; c Department of Health Data Science, University of Liverpool, Liverpool, United Kingdom; d Health Innovation Techcenter (HIT), Hospital Israelita Albert Einstein, São Paulo, Brazil; e Barão de Mauá University Center, Ribeirão Preto, SP, Brazil; f Centro Rosarino de Estudios Perinatales (CREP), Rosario, Argentina; g Department of Social Medicine, Ribeirão Preto Medical School, University of Sao Paulo, Ribeirão Preto, Brazil; h UNDP-UNFPA-UNICEF-WHO-World Bank Special Programme of Research, Development and Research Training in Human Reproduction (HRP), Department of Sexual and Reproductive Health and Research, World Health Organization, Geneva, Switzerland

**Keywords:** Early warning systems, Sepsis, Maternal sepsis, Early identification, Severe maternal outcome

## Abstract

**Background:**

Infections and sepsis are leading causes of morbidity and mortality in women during pregnancy and the post-pregnancy period. Using data from the 2017 WHO Global Maternal Sepsis Study, we explored the use of early warning systems (EWS) in women at risk of sepsis-related severe maternal outcomes.

**Methods:**

On April 27, 2023, we searched the literature for EWS in clinical use or research in obstetric populations. We calculated the proportion of women for whom each existing EWS identified them as at risk for developing severe maternal outcomes by infection severity (complications and severe maternal outcomes). Sensitivity, specificity, positive and negative likelihood ratios, diagnostic odds ratios, and J statistics were calculated to assess EWS performance. Machine learning was used to test the diagnostic potential of routine maternal sepsis markers.

**Findings:**

21 EWS were assessed in 2560 women from 46 countries with suspected or confirmed infections. The NICE Risk Stratification tool, Modified Shock Index, maternity Systemic Inflammatory Response Syndrome, and Early Maternal Infection Prompts scores had high sensitivity (88.1–97.5%) for identifying sepsis-related severe maternal outcomes. The quick Sequential Organ Failure Assessment (SOFA) in Pregnancy score and Obstetrically modified SOFA had high specificity (90.4–100%) for identifying women with sepsis-related severe maternal outcomes. Furthermore, combinations of sepsis markers had very low sensitivity and high specificity using machine learning.

**Interpretation:**

No score demonstrated enough diagnostic accuracy to be used alone to identify sepsis. However, obstetric—and sepsis-specific EWS performed better for early identification of maternal sepsis than non-obstetric and non-sepsis-specific scoring systems. There are limitations to applying EWS to real-world data, mainly due to the incompleteness of medical data that hinders EWS effectiveness. There is a need to continue developing and testing criteria for early identification of maternal sepsis.

**Funding:**

UNDP-UNFPA-UNICEF-WHO-World Bank Special Programme of Research, Development and Research Training in Human Reproduction (HRP), 10.13039/100004423WHO, Merck for Mothers, 10.13039/100000200U.S. Agency for International Development, 10.13039/100010269Wellcome Trust, and 10.13039/501100000272National Institute for Health and Care Research.

**Research in context**

**Evidence before this study**

On April 27, 2023, we searched MEDLINE, Scopus, Cochrane CENTRAL, and the WHO Clinical Trial Registry using the terms “pregnant OR postpartum women” AND “early warning system” AND “severe maternal outcome OR sepsis” in English from database inception. A 2019 review by Umar et al. reported on twelve obstetric EWS validation studies (published between January 1997 and March 2018) that linked high EWS scores to adverse obstetric outcomes. Additional evidence suggests that warning tools improve maternal and newborn care, leading to fewer complications through timely interventions. A few low-quality studies indicate that EWS may enhance obstetric care by increasing vital sign monitoring before caesarean delivery. However, results on EWS efficacy for maternal infections (like chorioamnionitis) are mixed, as they poorly predict sepsis and show low positive predictive values for severe morbidity and ICU admissions, with high false-positive rates that can impair care and cause alarm fatigue.

**Added value of this study**

This study evaluated 21 obstetric and non-obstetric, as well as sepsis-specific and non-sepsis-specific EWS, utilising clinical data from the medical records of women across 46 countries during and after pregnancy. It analyses how well different EWS can detect women at risk of severe maternal outcomes related to sepsis generally and in both high- and low-resource settings. None of the scores exhibited sufficient diagnostic accuracy for identifying sepsis-related severe maternal outcomes.

**Implications of all the available evidence**

Current insights from the Global Maternal Sepsis Study highlight that the most effective EWS for obstetric patients remains unclear. Nonetheless, using obstetric-specific EWS coupled with other clinical investigations can improve the recognition, assessment, and prompt treatment of women at risk of sepsis-related severe maternal outcomes. Further research should investigate alternative biological markers and diagnostic criteria for maternal sepsis and continue to optimise existing obstetric EWS to improve their accuracy.

## Introduction

Every year, approximately 287,000 women globally lose their lives due to pregnancy and childbirth complications, equating to almost 800 maternal deaths per day, or one every 2 min.^1^ Infections and sepsis have historically been linked to maternal deaths and continue to be leading causes of morbidity and mortality among women during and after pregnancy.^2^ The most recent global estimates of causes of maternal mortality suggest that 11% of maternal deaths have an acute infection or sepsis as the underlying cause.^3^ In addition, even when infection and sepsis are not the primary cause of death, they are a significant aggravating factor and contributing cause to many deaths attributed to other pregnancy-related complications (e.g., women with eclampsia who develop nosocomial pneumonia).^4^^,^^5^

Building on the sepsis-3 definition developed in 2016 by the Third Consensus on Sepsis,^6^^,^^7^ maternal sepsis is considered “a life-threatening condition defined as organ dysfunction resulting from infection during pregnancy, childbirth,^6^ postpartum, and post-abortion”.^8^ To operationalise the new definition of maternal sepsis, identification criteria that are simple to obtain, preferably bedside clinical signs, actionable, and applicable to high- and low-resource settings are required to identify women with possible severe maternal infections to enable prompt therapeutic action.^8^ By 2017, existing sepsis scores and warning signals, such as the Sequential Organ Failure Assessment (SOFA) score,^6^ quick SOFA (qSOFA),^9^ and Systemic Inflammatory Response Syndrome (SIRS)^10^ had not been validated among obstetric populations. Other warning signals used among pregnant and recently pregnant women, such as the Modified Early Obstetric Warning System (MEOWS) or the Irish Maternity Early Warning System (IMEWS), were not specific to infection or sepsis.^6^^,^^11^^,^^12^

In 2017, the World Health Organization (WHO) led the global maternal sepsis study and awareness campaign (GLOSS).^5^^,^^13^ GLOSS had three objectives: 1) to raise maternal sepsis awareness, 2) to investigate the frequency and management of pregnant or recently pregnant women admitted with suspected or confirmed infections globally, and 3) to explore the use of early warning systems (EWS) or severity markers in identifying women with potentially life-threatening infections and maternal sepsis. EWS are bedside tools used to evaluate hospitalised patients at risk of deterioration at an early stage. This paper focuses on the third objective of GLOSS, which hypothesised that EWS could accurately identify women at risk of developing sepsis-related severe maternal outcomes by flagging those who require early therapeutic interventions.

## Methods

### Study design

GLOSS was a facility-based, prospective, one-week inception cohort study accompanied by an awareness campaign; the protocol and initial findings have been published elsewhere.^5^^,^^13^^,^^14^ Between 28 November and 4 December 2017, GLOSS enrolled all women with suspected or confirmed infection admitted to or already hospitalised in any of the 713 participating facilities across 52 countries during any stage of pregnancy through 42 days after the end of pregnancy (abortion or childbirth). For this analysis, we included data on women from 446 facilities across 46 countries that collected individual data on the initial identification and management of suspected or confirmed infection. Individual data on women and their newborns were obtained from medical records, collected through a paper-based form, and later uploaded onto an online data management system developed for GLOSS. Individual-level data were collected for up to six weeks or until the woman was discharged, transferred outside the study area, or died. Individual forms gathered information about pregnancy status, clinical signs and symptoms, and laboratory markers upon enrolment (day 0, day −1, and day +1) into the study, management of the infection, complications, including any near-miss criteria (Supplementary file S1) during a hospital stay, and pregnancy and maternal outcomes.

### Ethics

Ethical approvals were obtained from WHO’s Ethics Review Committee (A65787) and each participating country according to local regulations. De-identified data was used for this analysis from GLOSS participants whose written informed consent or a waiver of written consent was obtained as required by local or national committees.

### Variable definitions

We employed the WHO maternal near-miss criteria, grounded in the Sequential Organ Failure Assessment (SOFA) score,^6^ to recognise organ dysfunction during and after pregnancy. These extensively validated criteria comprise clinical, management-based, and laboratory markers. Any woman meeting these criteria was considered to have organ dysfunction, and we harmonised our definition of maternal sepsis with the Sepsis-3 consensus to identify true positive cases related to pregnancy. Women with infection were assigned to three groups according to the severity of the infection during hospital stay.^14^ Group 1: infection-related severe maternal outcomes (a proxy for maternal sepsis) included women presenting with WHO near-miss criteria to define organ dysfunction or maternal death corresponding to the prespecified primary outcome of the GLOSS.^5^ Group 2: infections with complications included women with an invasive procedure to treat the source of infection (vacuum aspiration, dilatation, curettage, wound debridement, drainage [incision, percutaneous, culdotomy], laparotomy and lavage, other surgery), admission to intensive care unit or high dependency care, or transfer to another facility.^14^ The remaining population, group 3, was classified as having less severe infections.

### Early warning systems (EWS)

On April 23, 2023, we searched the literature for all EWS in use in obstetric populations. We used search terms “pregnant OR postpartum women” AND “early warning system” AND” severe maternal outcome OR sepsis”. The search terms are listed in Supplementary file S2.

EWS are based on physiological criteria (blood pressure, pulse rate, respiratory rate, temperature, and consciousness level) and may also include markers of organ dysfunction.^15^ Each score is calculated based on the deviation from the normal range or baseline assessment.

When one or more criteria are abnormal, a score is considered “triggered,” indicating at-risk patients who need a medical response.^15^ The scores were calculated based on vital signs and clinical and laboratory data from day −1, day 0, or day +1. They are calculated for women with complete data. The numerator represents the number of women with triggered scores out of the total for each group, including those with suspected or confirmed infections, complicated infections, severe maternal outcomes (SMO), hospitalisations over seven days, and ICU stays over three days.

### Statistical analyses

We conducted descriptive analyses of patient characteristics and assessed the performance of EWS to trigger for women across different infection severity groups during their hospital stay. We calculated sensitivity, specificity, positive and negative likelihood ratios, and diagnostic odds ratios (with 95% confidence intervals). The J-statistic, or Youden’s index, was used to measure the likelihood of positive score results.^16^ These analyses were carried out overall and separately for participants recruited in higher and lower income settings, with countries grouped based on income and region (Supplementary file S3).

The discriminatory results of the EWS were presented in a receiver operating characteristic (ROC) plot as an adapted Zombie Plot^17^ to visualise its efficacy in identifying women at risk of developing sepsis-related severe maternal outcomes. The plot compared sensitivity against specificity. It can be used to visualise how acceptable a scoring system's performance is. However, it is recognised that precise definitions of what makes an EWS acceptable or not are context specific.^17^

We calculated the percentages of missing data for women whose scores could not be calculated due to missing variables in their health records.

We also implemented machine learning (ML) techniques (Supplementary file S4) to investigate the diagnostic capacity of combinations of routine clinical and laboratory markers that define a diagnosis for sepsis as potential alternative criteria to scoring systems. To do so, we selected clinical and laboratory variables from the original database that define sepsis. The outcome, severe maternal outcome (SMO), was categorized as 'yes' or ‘no’. The data were split into a training set (80%) and a testing set (20%), with SMO distribution balanced across both. We created datasets with different variables combinations based on the importance of each variable, defined by attribute selection algorithms. We train nine classifiers in each dataset and evaluate their diagnostic accuracy using cross-fold validation. The performance metrics included sensitivity, specificity, false positive/negative rates, and area under the ROC curve. To identify the best classifiers, we set cut-off points. We used R programming language version 4.1.0 for the diagnostic accuracy analyses, Weka,^18^ SAS version 9.4, STATA version 14.2 (College Station, TX), and Microsoft Excel to perform the ML analyses.

### Role of the funding source

The funders of the study had no role in study design, data collection, data analysis, data interpretation, or writing of the report.

## Results

### Identification of EWS

**Fig. 1:**
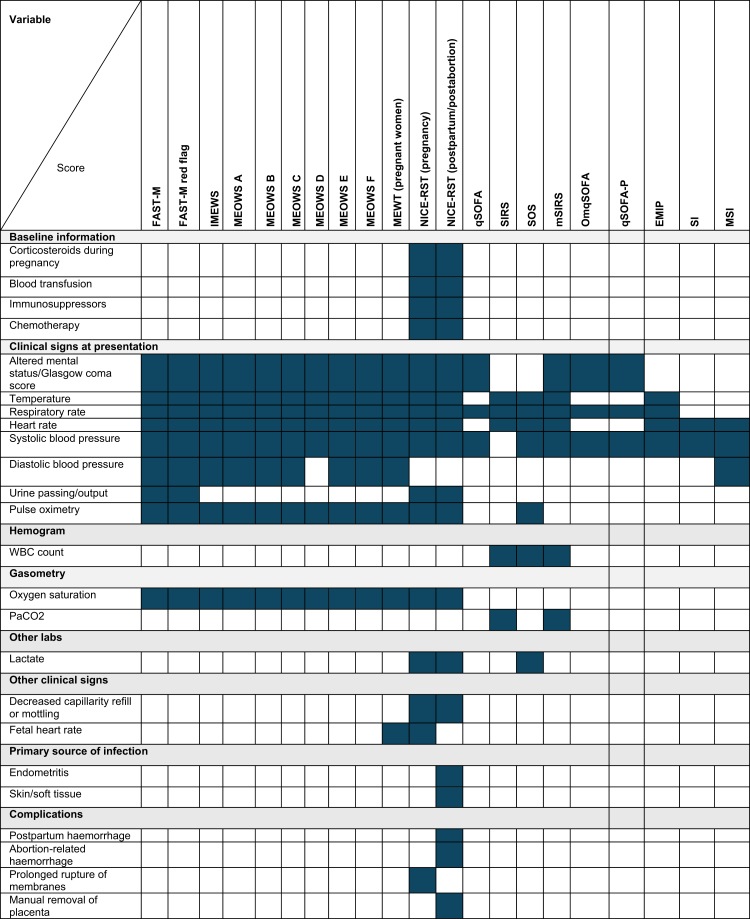
Early warning systems and composition of each score. Shadowed boxes mean that those variables are needed for the specific EWS. Shadowed boxes means that those variables are needed for the specific EWS. FAST-M: fluids, Antibiotics, Fluids, Antibiotics, source identification and control, transfer to an appropriate level of care, and ongoing monitoring of mother and neonate; FAST-M_RF: Fluids, Antibiotics, source identification and control, transfer to a proper level of care, and ongoing monitoring of mother and neonate red flags; IMEWS: Irish Maternity Early Warning System; MEOWS A-F: Modified early obstetric warning score; MEWT: Maternal Early Warning Trigger; NICE-RST-P: National Institute for Health and Care Excellence Risk Stratification Tool for pregnancy; NICE-RST-PP/PA: National Institute for Health and Care Excellence Risk Stratification Tool for postpartum or post-abortion; qSOFA: quick Sequential Organ Failure Assessment; SIRS: Systemic inflammatory response syndrome; SOS: Sepsis in Obstetrics Score; mSIRS: maternity systemic inflammatory response syndrome; OmqSOFA: Obstetrically modified quick Sequential Organ Failure Assessment; qSOFA-P: quick Sequential Organ Failure Assessment in Pregnancy; EMIP: Early Maternal Infection Prompt; SI: Shock Index; MSI: Modified Shock Index; mSIRS.

From the literature search, 28 EWS were in use or under investigation in the obstetric population. We excluded seven scores because some variables needed for those score calculations were unavailable in the GLOSS database (Supplementary file S5). Thus, 21 scores were evaluated in this study as early warning systems for developing sepsis-related severe maternal outcomes (Fig. 1 and Supplementary file S6).

### Patient characteristics

**Fig. 2:**
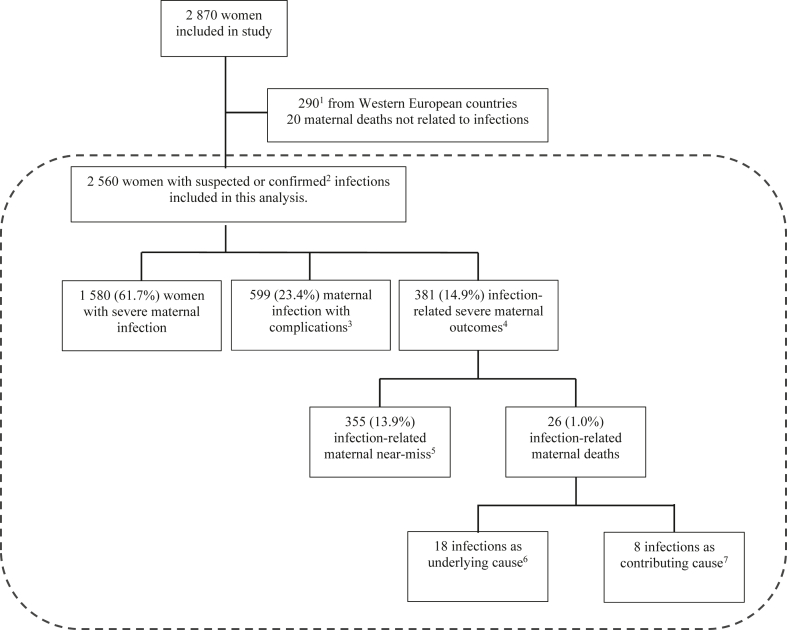
Study flowchart of included women by infection severity. The dotted line indicates women included in this analysis. ^1^290 women using a modified protocol in Western European countries (Belgium, Denmark, Italy, Spain, the Netherlands, and the United Kingdom) were excluded. ^2^Sources of infection were confirmed clinically, radiologically, or microbiologically. ^3^Maternal infections with complications were defined as women needing intensive care unit admission or invasive procedures to treat the source of infection or transfer. ^4^Infection-related severe maternal outcomes were defined as near miss or death. ^5^A proportion of women with infection-related severe maternal outcomes met at least one WHO near-miss criterium. ^6^Among the maternal deaths caused by an underlying infection, seven deaths were due to direct causes, five were due to abortion, and six were due to indirect causes. ^7^Among the maternal deaths with infection as a contributing cause were two deaths due to obstetric haemorrhage, one due to hypertensive disorder, one other direct cause, two due to indirect cause, and 2 with unknown cause.

Out of 2870 women who were enrolled in the one-week recruitment period of GLOSS, 2560 women with suspected or confirmed infections and available clinical data for day −1, 0, and +1 were classified into three groups: infection-related SMO, infections with complications, and less severe infections for this analysis. Of these, 1580 (61.7%) had a less severe maternal infection, 599 (23.4%) had a maternal infection with complications, and 381 (14.9%) had an infection-related SMO (Fig. 2). Most women (74.0%) were in the 20–35 age range, 53.2% were recently pregnant at enrolment, and about half (50.5%) arrived at the facility from their homes (Supplementary file S7).

### Missing values

Missing values were highest for partial pressure of carbon dioxide (PaCO2) (92.7%), lactate (93.4%), urine output (67.8%), pulse oximetry (66.8%), and 50.4% for both Glasgow Coma Score and temperature parameters overall for all participants. The frequency of missing values varied between day −1, day 0, and day +1 following enrolment into GLOSS. Most variables with substantially missing values corresponded to the day before and the first day after enrolment (day −1 and day +1) into GLOSS. There was fewer incomplete data on the day of enrolment (day 0) into GLOSS (Supplementary file S8).

### EWS performance

Overall, EWS were the most triggered for women admitted to ICU, followed by women with infection-related severe maternal outcomes, compared to women with infections with complications or prolonged hospital stays (Table 1). The NICE Risk Stratification tool (NICE-RST) in postpartum or postabortion women (PP/PA) and the Modified Shock Index (MSI) were the two most triggered scores overall. The MSI identified 94.3% (1994/2114), and the NICE-RST-PP/PA identified 83.1% (354/426) of all women with suspected or confirmed infection. The MSI identified 94.7% (780/824) and the NICE-RST-PP/PA 91.7% (210/229) of women with infection with complications. The NICE-RST-PP/PA identified 97.7% (83/85), and the MSI identified 95.7% (313/327) of women with SMO as being at risk of developing sepsis. Similarly, the MSI and NICE-RST-PP/PA scores triggered for 94.1% (704/748) and 90.7% (194/214) of women who had a hospital stay of over a week, and 95.4% (165/173) and 90.6% (48/53) of women admitted to the intensive care unit (ICU) for at least three days, respectively. The EMIP also triggered for many women, identifying 81.5% (729/895) women with suspected or confirmed infection, 86.2% (313/363) with infections with complications, and 88.1% (156/177) with SMO to be at risk for sepsis. Generally, the obstetric-modified versions of the scores were triggered in more women with severe maternal outcomes compared to the non-obstetric scores. For example, mSIRS triggered in 89.2% (116/130) of women with SMO and in 93.5% (72/77) of women admitted to ICU, compared to SIRS, which triggered in only 62.9% (161/256) of women with SMO and 66.7% (96/144) of women admitted to ICU.

**Table 1:** Distribution of triggered early warning score systems for all women, with complicated infections, with severe maternal outcomes, with more than seven days of hospitalization, and with more than two days in the intensive care unit (ICU) (N = 2560).

Early warning system	All women with suspected or confirmed infections (N = 2560)	Infections with complications (N = 980)	Severe maternal outcomes related to infections (N = 381)	Length of hospital stay > 7 days (N = 868)	ICU admission ≧ 3 days (N = 182)
Obstetric scores—not sepsis specific					
FAST-M	343/1154 (29.7%)	168/447 (37.6%)	89/165 (53.9%)	124/398 (31.2%)	59/95 (62.1%)
FAST-M red flag	324/1532 (21.2%)	179/614 (29.1%)	115/254 (45.3%)	145/559 (25.9%)	72/140 (51.4%)
IMEWS	1214/1870 (64.9%)	555/764 (72.6%)	249/303 (82.2%)	472/674 (70.0%)	140/158 (88.6%)
MEOWS A	952/1858 (51.2%)	447/761 (58.7%)	220/302 (72.9%)	381/669 (57.0%)	124/158 (78.5%)
MEOWS B	967/1868 (51.8%)	449/764 (58.8%)	224/303 (73.9%)	375/673 (55.7%)	127/158 (80.4%)
MEOWS C	992/1869 (53.1%)	459/764 (60.1%)	227/303 (74.9%)	385/288 (57.2%)	128/158 (81%)
MEOWS D	384/1892 (20.3%)	236/772 (30.6%)	154/309 (49.8%)	186/678 (27.4%)	82/158 (51.9%)
MEOWS E	405/1870 (21.7%)	238/764 (31.1%)	150/303 (49.5%)	187/674 (27.7%)	83/158 (52.5%)
MEOWS F	695/1866 (37.2%)	362/761 (47.6%)	197/302 (65.2%)	302/672 (44.9%)	111/157 (70.7%)
MEWT (pregnant women)	59/749 (7.9%)	39/189 (20.6%)	28/91 (30.8%)	27/218 (12.4%)	20/58 (34.4%)
SOS	75/1957 (3.8%)	57/791 (7.2%)	49/315 (15.6%)	38/704 (5.4%)	18/161 (11.2%)
Obstetric scores—sepsis specific					
NICE-RST (Postpartum/Postabortion)	354/426 (83.1%)	210/229 (91.7%)	83/85 (97.7%)	194/214 (90.7%)	48/53 (90.6%)
NICE-RST (Pregnant Women)	317/517 (55.5%)	86/132 (65.2%)	43/64 (67.2%)	100/169 (59.2%)	33/41 (80.5%)
mSIRS	437/625 (69.9%)	206/254 (81.1%)	116/130 (89.2%)	226/307 (73.6%)	72/77 (93.5%)
OmqSOFA (pregnant women)	126/797 (15.8%)	78/323 (24.1%)	53/166 (31.9%)	56/376 (14.9%)	39/89 (43.8%)
qSOFA-P (pregnant women)	42/730 (5.75%)	31/287 (10.8%)	31/144 (21.5%)	19/348 (5.5%)	19/79 (24.1%)
EMIP (pregnant women)	729/895 (81.5%)	313/363 (86.2%)	156/177 (88.1%)	337/404 (83.4%)	87/98 (88.8%)
Scores –not obstetric or sepsis specific					
qSOFA	317/1711 (18.5%)	166/683 (24.3%)	96/270 (35.6%)	122/605 (20.2%)	55/148 (37.2%)
SIRS	554/1505 (36.8%)	286/614 (46.6%)	161/256 (62.9%)	236/547 (43.1%)	96/144 (66.7%)
SI	204/857 (23.8%)	87/326 (26.7%)	47/151 (31.1%)	96/399 (24.1%)	21/81 (25.9%)
MSI	1994/2114 (94.3%)	780/824 (94.7%)	313/327 (95.7%)	704/748 (94.1%)	165/173 (95.4%)

For all calculations, the numerator represents the number of women for whom the EWS was triggered, and the denominator represents the number of women included in any given group whom the EWS could be measured given data available. FAST-M: fluids, Antibiotics, Fluids, Antibiotics, source identification and control, transfer to an appropriate level of care, and ongoing monitoring of mother and neonate; FAST-M_RF: Fluids, Antibiotics, source identification and control, transfer to a proper level of care, and ongoing monitoring of mother and neonate red flags; IMEWS: Irish Maternity Early Warning System; MEOWS A-F: Modified early obstetric warning score; MEWT: Maternal Early Warning Trigger; NICE-RST-P: National Institute for Health and Care Excellence Risk Stratification Tool for pregnancy; NICE-RST-PP/PA: National Institute for Health and Care Excellence Risk Stratification Tool for postpartum or post-abortion; qSOFA: quick Sequential Organ Failure Assessment; SIRS: Systemic inflammatory response syndrome; SOS: Sepsis in Obstetrics Score; mSIRS: maternity systemic inflammatory response syndrome; OmqSOFA: Obstetrically modified quick Sequential Organ Failure Assessment; qSOFA-P: quick Sequential Organ Failure Assessment in Pregnancy; EMIP: Early Maternal Infection Prompt; SI: Shock Index; MSI: Modified Shock Index.

### Diagnostic accuracy of EWS

Our testing for diagnostic accuracy of EWS (Table 2) to identify women at risk for sepsis-related SMOs showed that while the NICE-RST-PP/PA and MSI both had high sensitivities (97.6% and 95.7%) among women with infection-related SMO, their specificities was very low, only 11.8% and 5.9%, with diagnostic odds ratios (DOR) of 5.56 (95% CI 1.25–24.67) and 1.41 (95% CI 0.74–2.7), respectively. In contrast, the OmqSOFA had a low sensitivity of 38.0% and a high specificity of 90.4% with a DOR of 5.79 (95% CI 3.12–10.74). The qSOFA-P showed low sensitivity (21.6%) but high specificity (100%) for identifying women at risk of sepsis-related SMOs.

**Table 2:** Diagnostic accuracy for sepsis-related severe maternal outcomes (near-miss or death) (N = 2560).

Early warning system	Sensitivity	Specificity	Positive Likelihood Ratio (95% CI)	Negative Likelihood Ratio (95% CI)	Diagnostic Odds Ratio (95% CI)	J statistic [Youden’s Index] (95% CI)
Obstetric scores—not sepsis specific						
FAST-M	53.9	72.0	1.92 (1.52–2.43)	0.64 (0.53–0.77)	3.01 (2.01–4.50)	0.26
FAST-M red flag	45.3	82.2	2.55 (1.96–3.30)	0.67 (0.59–0.75)	3.83 (2.65–5.52)	0.27
IMEWS	82.2	33.6	1.24 (1.14–1.35)	0.53 (0.40–0.70)	2.34 (1.64–3.32)	0.16
MEOWS A	72.8	50.5	1.47 (1.31–1.65)	0.54 (0.44–0.66)	2.74 (2.01–3.75)	0.23
MEOWS B	73.9	51.2	1.51 (1.35–1.70)	0.51 (0.41–0.63)	2.97 (2.17–4.07)	0.25
MEOWS C	74.9	49.7	1.49 (1.33–1.66)	0.50 (0.41–0.63)	2.95 (2.15–4.05)	0.25
MEOWS D	49.8	82.3	2.81 (2.24–3.53)	0.61 (0.54–0.69)	4.62 (3.33–6.40)	0.32
MEOWS E	49.5	80.9	2.59 (2.08–3.23)	0.62 (0.55–0.70)	4.16 (3.01–5.74)	0.30
MEOWS F	65.2	64.1	1.81 (1.57–2.10)	0.54 (0.46–0.64)	3.34 (2.47–4.53)	0.29
MEWT (Pregnant Women)	30.8	88.8	2.74 (1.45–5.18)	0.78 (0.67–0.91)	3.52 (1.63–7.59)	0.20
Obstetric scores—sepsis specific						
NICE-RST (Postpartum/Postabortion)	97.6	11.8	1.11 (1.03–1.19)	0.20 (0.05–0.84)	5.56 (1.25–24.67)	0.09
NICE-RST (Pregnant Women)	67.2	36.8	1.06 (0.83–1.36)	0.89 (0.56–1.43)	1.19 (0.58–2.44)	0.04
mSIRS	89.2	27.4	1.23 (1.09–1.39)	0.39 (0.22–0.7)	3.13 (1.58–6.18)	0.17
OmqSOFA (pregnant women)	38.0	90.4	3.97 (2.36–6.68)	0.69 (0.6–0.78)	5.79 (3.12–10.74)	0.28
qSOFA-P (pregnant women)	21.6	100	bINF	0.78 (0.72–0.85)	bINF	0.22
EMIP (pregnant women)	88.1	15.6	1.04 (0.96–1.13)	0.76 (0.45–1.28)	1.37 (0.75–2.51)	0.04
Scores—not obstetric or sepsis specific						
qSOFA^a^	35.6	83.1	2.10 (1.61–2.74)	0.78 (0.70–0.86)	2.70 (1.89–3.87)	0.19
SIRS^a^	62.9	65.1	1.80 (1.52–2.13)	0.57 (0.48–0.68)	3.16 (2.26–4.41)	0.28
SI	31.1	77.1	1.36 (0.95–1.95)	0.89 (0.78–1.02)	1.53 (0.93–2.50)	0.08 (−0.06 to 0.22)
MSI	95.7	5.9	1.02 (0.99–1.05)	0.72 (0.39–1.34)	1.41 (0.74–2.7)	0.02 (−0.03 to 0.06)

a For women enrolled in the study during their stay in the intensive care unit (ICU).

b INF = Impossible to calculate, for the sensitivity or 1-specificity values correspond to 100% of the sample with or without sepsis. FAST-M: fluids, Antibiotics, Fluids, Antibiotics, source identification and control, transfer to an appropriate level of care, and ongoing monitoring of mother and neonate; FAST-M_RF: Fluids, Antibiotics, source identification and control, transfer to a proper level of care, and ongoing monitoring of mother and neonate red flags; IMEWS: Irish Maternity Early Warning System; MEOWS A-F: Modified early obstetric warning score; MEWT: Maternal Early Warning Trigger; NICE-RST-P: National Institute for Health and Care Excellence Risk Stratification Tool for pregnancy; NICE-RST-PP/PA: National Institute for Health and Care Excellence Risk Stratification Tool for postpartum or post-abortion; qSOFA: quick Sequential Organ Failure Assessment; SIRS: Systemic inflammatory response syndrome; SOS: Sepsis in Obstetrics Score; mSIRS: maternity systemic inflammatory response syndrome; OmqSOFA: Obstetrically modified quick Sequential Organ Failure Assessment; qSOFA-P: quick Sequential Organ Failure Assessment in Pregnancy; EMIP: Early Maternal Infection Prompt; SI: Shock Index; MSI: Modified Shock Index.

Among the high and upper-middle-income countries, 105 women had infection-related SMO (Supplementary file S9). The NICE-RST-PP/PA (sensitivity 95.65%, DOR 3.38 [95% CI 0.38–29.95]) and EMIP (sensitivity 84.62%, DOR 1.43 [95% CI 0.55–3.71]) were the two scores with the highest sensitivity, whereas the SOS (specificity 99.95%, very high DOR 22 [95% CI 2.81–172.14]) and qSOFA-P (specificity 100%, very high PPV 1.0 [95% 0.69–1.0]) had the highest specificities. In low and lower-middle-income countries, there were 276 women with infection-related SMO. The NICE-RST-PP/PA (sensitivity 98.39%, high DOR 7.63 [95% CI 0.10–60.61]) and mSIRS (sensitivity 91.76%, DOR 4.29 [95% CI 1.69–10.86]) had high sensitivity, whereas the SOS (specificity 97.82%, high DOR 9.12 [95% CI 3.98–20.87]) and qSOFA-P (specificity 100%, high PPV 1.0 [95% CI 0.84–1.0]) were highly specific scores.

### Efficacy of EWS

**Fig. 3:**
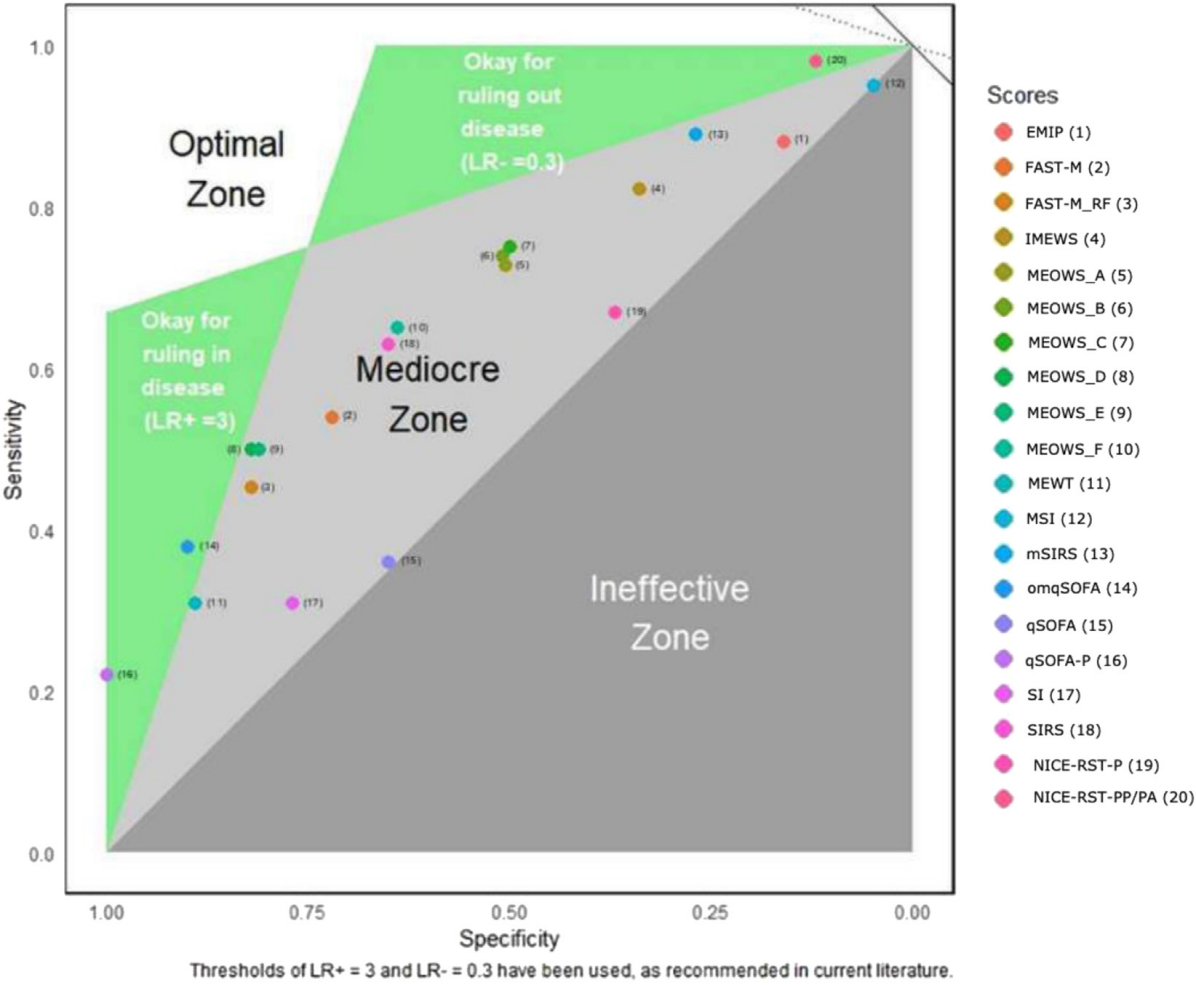
Receiver operating characteristic curve (ROC) Plot showing five zones of the zombie plot (ROC plot divided into zones) of early warning systems efficacy for identifying the development of sepsis-related severe maternal outcomes. The white and green zones form a slender, boomerang-shaped area of acceptable efficacy in the upper left corner. If the sensitivity and specificity values of an EWS (and their 95% confidence interval) lie within the boomerang-shaped area, then that EWS has acceptable diagnostic credibility. LR+: Positive likelihood ratio; LR-: Negative Likelihood ratio; EMIP: Early Maternal Infection Prompt; FAST-M: Fluids, Antibiotics, Fluids, Antibiotics, Source identification and control, Transfer to an appropriate level of care, and ongoing Monitoring of mother and neonate; FAST-M_RF: Fluids, Antibiotics, Source identification and control, Transfer to a proper level of care, and ongoing Monitoring of mother and neonate red flags; IMEWS: Irish Maternity Early Warning System; MEOWS A–F: Modified early obstetric warning score; MEWT: Maternal Early Warning Trigger; MSI: Modified Shock Index; mSIRS: maternity systemic inflammatory response syndrome; omqSOFA: Obstetrically modified quick Sequential Organ Failure Assessment; qSOFA: quick Sequential Organ Failure Assessment; SI: Shock Index; SIRS: Systemic inflammatory response syndrome; NICE-RST-P: National Institute for Health and Care Excellence Risk Stratification Tool for pregnancy; NICE-RST-PP/PA: National Institute for Health and Care Excellence Risk Stratification Tool for postpartum or post-abortion.

When assessing the efficacy of EWS in identifying women at risk of sepsis, the NICE-RST-PP/PA score fell in the “acceptable efficacy” zone for ruling out sepsis (Fig. 3). The qSOFA-P and OmqSOFA scores fell in the acceptable efficacy zone for ruling in sepsis. None of the EWS fell in the optimal zone, with most EWS falling in the mediocre zone, considered suboptimal for detecting or excluding the development of sepsis among women with SMOs.

### Machine learning analysis

The trained models that presented the best diagnostic metrics use statistical approaches such as Naive Bayes and Logistic Regression. Modelling combinations of sepsis markers (clinical and laboratory variables) obtained on day 0 (model 2) and day +1 (model 3) revealed notably high specificity (0.97–1.00), robust area under the receiver operating characteristic (AUROC) (0.75–0.85), and high odds ratio values (14.61–30.2). However, the combinations also had low sensitivity (Supplementary file S10).

## Discussion

We aimed to assess the efficacy of diagnostic criteria for sepsis using existing EWS, drawing upon data from 2560 women participating in the GLOSS. Our analysis centred on the performance of 21 EWS in identifying pregnant or recently pregnant women at risk of developing severe maternal outcomes related to sepsis, using routine clinical data obtained during hospitalisation for suspected or confirmed infections.

Our analysis found that obstetric- and sepsis-specific EWS performed better for early identification of maternal sepsis. At the same time, the non-obstetric and non-sepsis scores, such as qSOFA and SIRs, performed poorly. Researchers have reported that non-obstetric qSOFA is a poor predictor of adverse outcomes in pregnant patients with sepsis.^19^ Pregnancy-specific modifications, or “qSOFA-P,” significantly improve the ability of the score to predict severe maternal morbidity.^19^ The SIRS criteria has been reported to over-trigger in pregnant women without clinical significance.^20^ In our study, the mSIRS performed better in detecting infection-related SMOs than the non-obstetric SIRS score, with higher sensitivity (89.2% vs 62.9%). The mSIRS likely incorporates adjustments that make it more tailored to the physiological changes in pregnancy, reducing the likelihood of over-flagging women who are not clinically deteriorating. The overwhelming evidence that vital sign ranges change during pregnancy in a gestation-specific manner justifies the development and use of obstetric-specific criteria to diagnose maternal sepsis.^21^ Nevertheless, attention must be directed towards optimising obstetric-specific sepsis scores by implementing evidence-based calibration of vital sign thresholds within obstetric early warning score systems that can be globally applicable.^22^

An effective EWS for maternal sepsis should accurately confirm or rule out the condition, enabling prompt identification and escalation of care.^23^ Clinical application of EWS may necessitate a trade-off between sensitivity and specificity when selecting the ideal EWS for different clinical contexts. For instance, the NICE-RST-PP/PA has high sensitivity but low specificity and is recommended by the National Institute for Health and Care Excellence for in-patient use in the United Kingdom.^24^ As the NICE-RST-PP/PA incorporates a serum lactate measurement, it would be considered a good score for use in high-resource settings where point-of-care devices and better laboratory capacity exist. With equally high sensitivity, the MSI score would be a better fit for low-resource settings, where a single reading using a digital blood pressure machine provides the required vital signs.^25^ In this study, EWS demonstrated consistent performance in higher and lower-resourced settings. For example, the NICE-RST-PP/PA, mSIRS, and EMIP all had high sensitivity (but low specificity) in both high and low-resource settings. The OmqSOFA and qSOFA scores had high specificity (with low sensitivity) in both settings. However, the GLOSS study observed significant differences in infection frequency and maternal outcomes, resource availability variations, and differing admission thresholds to identify or treat complications between high- and low-income settings.^14^

Finding the right balance between the sensitivity and specificity of EWS is crucial for accurate patient care in both high- and low-resource settings. Although an ideal EWS should have high sensitivity to detect sepsis promptly and prevent severe maternal outcomes, this can result in many false positive cases, which could be wasteful. Using high-specificity EWS to identify maternal sepsis cases and accurately avoid missed diagnoses may be preferred in some settings. This approach can help prevent overwhelming healthcare services and ensure proper care for women with other medical conditions in settings with limited capacity. On the other hand, an EWS with high sensitivity but lower specificity may lead to numerous referrals for costly diagnostic procedures, also overwhelming healthcare systems. On the other hand, an EWS with high specificity but low sensitivity might result in missed cases and delayed treatment. The optimal strategy may involve using different tests for different stages of diagnosis or employing a combination of high sensitivity and specificity tests to balance accuracy and efficiency. For example, the measurement of blood lactate forms a key part of sepsis management and risk stratification in current international guidelines, where, in practice, a positive qSOFA (>2) score calculation is routinely followed up with a serum lactate test. This sequential testing has been proven to be a good predictor of poor outcomes in high-income countries,^26^ where point-of-care lactate testing devices make the result rapidly available. A current multi-country study is underway to determine the additional diagnostic value of blood lactate in addition to conventional vital signs in maternal patients in low-resource settings.^25^

Our analysis identified no ideal EWS from the existing tools published or using machine learning to optimise a new score. It is, therefore, critical to consider the intended use and context in which the tool will be used before concluding its usefulness. This consideration is crucial in selecting the most appropriate EWS for a given clinical context. For instance, the FAST-M score was designed with high thresholds for the vital sign triggers, which would reduce false-positive results. This was because of concerns in low-resource settings where triggering a bundle of care could rapidly overwhelm the limited provider capacity, with subsequent trigger fatigue if the tool were regularly being inappropriately triggered in women who are not at risk of adverse outcomes.^27^ Meanwhile, other scores may only prompt further investigation with fewer consequences for over-triggering. Some scores are tailored for specific populations or clinical scenarios. Our analysis compared all scores, regardless of their intended purpose, to patient data retrieved from hospital records at specific time points without considering the condition's severity at study entry. The limitation of any tool is that it will need to be used alongside clinical judgement and experience, including communication with the patient or their caregivers, who may also offer insights into their changing condition and with careful monitoring of trends over time. Clinicians need to be aware of these limitations in the available tools, so they are willing to reconsider a diagnosis if the clinical condition changes.

Our study retrospectively applied EWS to GLOSS data. The real-world use of EWS depends on the ease of use of each score and the availability of the various components for calculation and interpretation. However, it is crucial to note that we did not assess the barriers and facilitators of using EWS when infection is first suspected or confirmed. We found that essential patient data, such as the Glasgow Coma Scale (GCS) and temperature, was absent for over 50% of participants. Out of the 21 scores analysed in this study, 17 required the inclusion of the GCS and 16 need temperature readings in their score calculations. The high proportion of missing data for key clinical parameters significantly hampers the accuracy and reliability of evaluating the performance of EWS. Additionally, the diagnostic capability of these EWS for detecting severe adverse outcomes is compromised, given that these scores rely on vital signs of physiological function crucial to life. The lack of specific data may indicate insufficient monitoring (a healthcare quality factor), or healthcare providers may use different vital sign combinations to detect sepsis-related deterioration (a resource-limiting factor). For instance, the high percentage of missing data for PaCO2, serum lactate, pulse oximetry, and urine output could indicate differences in resource availability for medical equipment or infrastructure needed to measure these parameters. This further highlights the need for the use of context-appropriate EWS. The missing parameters in this study meant we could only apply the scores to the maternal patients whose data was complete. Our findings suggest that using obstetric-specific EWS is beneficial for identifying SMOs related to sepsis. However, recent studies have produced conflicting results. Bauer et al. found that non-obstetric sepsis screening tools were more effective during pregnancy and up to 3 days after childbirth compared to pregnancy-adjusted sepsis screening tools.^28^ Therefore, establishing a standardised approach for assessing sepsis risk in obstetric patients is crucial, given the ample evidence supporting the effectiveness and benefits of EWS in preventing avoidable adverse outcomes in the general population.^29^ EWS offer broader advantages by aiding clinical decision-making, particularly in settings with limited access to advanced diagnostic tools. By tracking changes in a patient's condition over time and promoting thorough record-keeping, EWS can contribute to improved quality and consistency of care.^30^ Implementing EWS can also help prioritise care for critically ill women, ensuring that those in immediate need receive attention despite limited resources. EWS can be easily taught to healthcare workers, empowering staff in resource-limited settings to recognise and respond to signs of sepsis, particularly in the absence of experienced clinicians.^31^ Overall, integrating EWS into the care of obstetric patients can be a valuable strategy for effectively managing and preventing maternal sepsis.

This study provides a real-world application and assessment of EWS using routine clinical data from medical records of pregnant or recently pregnant women with suspected or confirmed infections on a global scale. The data collected from women hospitalised in low, middle, and high-income countries provides substantial evidence on preventive measures against life-threatening complications related to infection. As no EWS was sufficiently accurate in our study, it suggests there is a potential gap in EWS for the maternal population that future research into new or refined scores should aim to address.

The analysis has limitations, including data collection at only three-time points focused on the time of confirmation or first suspicion of infection. This necessitates further high-quality studies to broadly explore the accuracy of EWS in identifying infection-related severe maternal outcomes and sepsis in the obstetric population. The generalizability of the findings to all contextual and clinical settings is limited, mainly due to variations in resource availability and healthcare practices. These limitations highlight the need for further research and improvement in the early identification of maternal sepsis.

There is a pressing need to continue developing and testing criteria for early identification of maternal sepsis. With the limited accuracy of existing tools, more research is required to test biological markers and other diagnostic criteria. Without the ideal EWS, tools will need to be selected to optimise performance based on the use case's requirements and broader clinical context.

## Contributors

JPS, MB, and VB conceptualised this analysis. JPS wrote an earlier draft of this manuscript with contributions from VB. YC wrote the final draft of this paper, conducted the literature review, and led all analyses with support from DL, VB, and MB. AH performed the statistical analysis with support from LB. LOC conducted the machine learning analyses. AH, LOC, LB, EA, CC, JPS, MB, VB, and DL contributed to the final manuscript. All authors and research group members approved the final version for publication. The named authors alone are responsible for the views expressed in this publication and do not necessarily represent the decisions or the policies of the UNDP-UNFPA-UNICEF-WHO-World Bank Special Programme of Research, Development and Research Training in Human Reproduction (HRP) or the World Health Organization (WHO) or any of the other affiliated institutions.

## Data sharing statement

Following the WHO policy for sharing and reusing research data, the analytic datasets containing individual participant-level data from this multi-country study data are made available under a data share agreement between the requester or requesting institution and the study institutions. Requesting these data may be made to the editorial board by emailing srhmph@who.int, a concept paper containing the intended use of data and individual contact information.

## Declaration of interests

The authors declare no competing interests. The collaboration between the Human Reproduction Programme, the Department of Sexual and Reproductive Health and Research, and Merck for Mothers is governed by a bilateral agreement.
