# Supplementary material for: Early warning systems for identifying severe maternal outcomes: findings from the WHO global maternal sepsis study
Source: eClinicalMedicine. 2024 Dec 6;79:102981. doi: 10.1016/j.eclinm.2024.102981 (PMC11667637; doi:10.1016/j.eclinm.2024.102981)
Supplement: Supplementary files S1–S11 [file mmc1.docx]

Table S1. WHO criteria for maternal near miss^4^

| CLINICAL CRITERIA |  |
| --- | --- |
| - Acute Cyanosis - Gasping - Breathing rate > 40 or < 6 per minute - Shock - Oliguria unresponsive to fluids or diuretics - Loss of consciousness for ≧ 12 h | - Unconscious, no pulse/heartbeat - Uncontrolled convulsions/total paralysis - Jaundice concomitantly with preeclampsia - Coagulation disorders/clotting failure |
| LABORATORY CRITERIA |  |
| - Oxygen saturation < 90% for > 60 minutes - PaO2/FiO2 < 200mmHg - Creatinine ≧ 300mmol/l or ≧ 3.5 mg/dl - Bilirubin > 100 mmol/l or ≧6.0 mg/dl | - pH < 7.1 - Lactate > 5 - Acute thrombocytopenia (< 50,000 platelets) - Unconscious, presence of glucose and ketoacidosis in urine |
| MANAGEMENT CRITERIA |  |
| - Use of continuous vasoactive drug - Postpartum or post abortion hysterectomy due to infection or haemorrhage - Blood transfusion ≧ 5 units or red cell | - Intubation and ventilation for a period >= 60 minutes, unrelated to anaesthesia - Dialysis for treatment of acute renal failure - Cardiopulmonary resuscitation (CPR) |

S2: Literature search strategy: MEDLINE (PubMed) example

| Search set | Search term |
| --- | --- |
| 1 | "Sepsis"[MeSH Terms:noexp] OR "Sepsis"[Text Word] OR "Sepsis"[Title/Abstract] OR "shock, septic"[MeSH Terms] OR "infection*"[MeSH Terms] OR "infection*"[Text Word] OR "infection*"[Title/Abstract] OR "infectious"[Text Word] |
| 2 | "early warning system"[MeSH] OR "severity of illness index"[MeSH] OR "simplified acute physiology score” [MeSH] OR “APACHE” [MeSH] OR “early warning score” [MeSH] OR “warning signal” [TextWord] OR “warning scores” [TextWord] OR “severity marker” [TextWord] |
| 3 | "Pregnant women"[MeSH Terms] OR "pregnant woman"[Text Word] OR "pregnan*"[MeSH Terms] OR "postpartum period"[MeSH Terms] OR "puerperium"[Text Word] OR "peripartum period"[MeSH Terms] OR "peripartum"[Text Word] OR "gravidity"[MeSH Terms] OR "gravidity"[Text Word] OR "abortion, spontaneous"[MeSH Terms] |
| 4 | 1 and 2 and 3 |
| 5 | "severe"[All Fields] AND ("maternally"[All Fields] OR "maternities"[All Fields] OR "maternity"[All Fields] OR "mothers"[MeSH Terms] OR "mothers"[All Fields] OR "maternal"[All Fields]) AND ("outcome"[All Fields] OR "outcomes"[All Fields]) |
| 6 | 2 and 5 |
| 7 | 4 OR 6 |

Search databases

- MEDLINE (PubMed)
- Scopus
- Cochrane CENTRAL
- WHO International Clinical Trial Registry Platform

S3: GLOSS list of countries by income and region (created October 2017)

|  | AFRO | AMRO | EMRO | EURO | SEARO | WPRO |
| --- | --- | --- | --- | --- | --- | --- |
| LIC + LMIC  (low income and lower middle income)  31 countries | 1. Benin (n=61) 2. Burkina Faso(n=39) 3. Cameroon (n=41) 4. Ethiopia (n=59) 5. Ghana (n=88) 6. Kenya (n=55) 7. Malawi (n=66) 8. Mali (n=40) 9. Moçambique (n=34) 10. Nigeria (n=66) 11. Senegal (n=50) 12. Zimbabwe (n=52) | 1. Bolivia (n=42) 2. Honduras(n=52) 3. Nicaragua (n=125) | 1. Afghanistan(n=43) 2. Egypt (n=10) 3. Morocco (n=125) 4. Pakistan (n=69) 5. Sudan (n=75) | 1. Kyrgyzstan (n=45) 2. Republic of Moldova (n=48) 3. Tajikistan (n=79) | 1. India (n=35) 2. Myanmar (n=34) 3. Nepal (n=40) 4. Sri Lanka (n=68) | 1. Cambodia (n=9) 2. Mongolia (n=36) 3. Philippines (n=33) 4. Vietnam (n=44) |
| UMIC + HIC (upper middle and high income)  15 countries | 1. South Africa(n=67) | 1. Argentina (n=84) 2. Brazil (n=48) 3. Colombia (n=78) 4. Ecuador (n=53) 5. Guatemala (n=60) 6. Mexico (n=217) 7. Peru (n=29) 8. Uruguay (n=42) | 1. Lebanon (n=5) | 1. Kazakhstan (n=137) 2. Lithuania (n=36) 3. Romania (n=7) 4. Slovakia (n=16) | 1. Thailand (n=18) |  |

S4: Machine learning methodology

|  |  |  |  |  |  |  |  |  |  |  |  |  |  |  |  |  |  |  |  |  |  |  |  |  |  |  |  |  |
| --- | --- | --- | --- | --- | --- | --- | --- | --- | --- | --- | --- | --- | --- | --- | --- | --- | --- | --- | --- | --- | --- | --- | --- | --- | --- | --- | --- | --- |
|  |  |  |  |  |  |  |  |  |  |  | Original database  N=2,560 | | | | | | | |  |  |  |  |  |  |  |  |  |  |
|  |  |  |  |  |  |  |  |  |  |  |  |  |  |  |  |  |  |  |  |  |  |  |  |  |  |  |  |  |
|  |  |  |  |  |  |  |  |  |  |  |  |  |  |  |  |  |  |  |  |  |  |  |  |  |  |  |  |  |
|  |  |  |  |  |  |  |  |  |  |  |  |  |  |  |  |  |  |  |  |  |  |  |  |  |  |  |  |  |
|  |  |  |  |  |  |  |  |  |  |  |  |  |  |  |  |  |  |  |  |  |  |  |  |  |  |  |  |  |
|  |  |  |  |  |  | Training dataset (80%)  N=2948 | | | | | | | |  |  | Test dataset (20%)  N=512 | | | | | | | |  |  |  |  |  |
|  |  |  |  |  |  |  |  |  |  |  |  |  |  |  |  |  |  |  |  |  |  |  |  |  |  |  |  |  |
|  |  |  |  |  |  |  |  |  |  |  |  |  |  |  |  |  |  |  |  |  |  |  |  |  |  |  |  |  |
|  |  |  |  |  |  |  |  |  |  |  |  |  |  |  |  |  |  |  |  |  |  |  |  |  |  |  |  |  |
|  |  |  |  |  |  |  |  |  |  |  |  |  |  |  |  |  |  |  |  |  |  |  |  |  |  |  |  |  |
|  |  |  |  |  |  | Feature selection  (10 approaches) | | | | | | | |  |  |  |  |  |  |  |  |  |  |  |  |  |  |  |
|  |  |  |  |  |  |  |  |  |  |  |  |  |  |  |  |  |  |  |  |  |  |  |  |  |  |  |  |  |
|  |  |  |  |  |  |  |  |  |  |  |  |  |  |  |  |  |  |  |  |  |  |  |  |  |  |  |  |  |
|  |  |  |  |  |  |  |  |  |  |  |  |  |  |  |  |  |  |  |  |  |  |  |  |  |  |  |  |  |
|  |  |  |  |  |  |  |  |  |  |  |  |  |  |  |  |  |  |  |  |  |  |  |  |  |  |  |  |  |
|  |  |  |  |  |  | 3 original +  3 complete databases +  26 feature selected databases | | | | | | | |  |  |  |  |  |  |  |  |  |  |  |  |  |  |  |
|  |  |  |  |  |  |  |  |  |  |  |  |  |  |  |  |  |  |  |  |  |  |  |  |  |  |  |  |  |
|  |  |  |  |  |  |  |  |  |  |  |  |  |  |  |  |  |  |  |  |  |  |  |  |  |  |  |  |  |
|  |  |  |  |  |  |  |  |  |  |  |  |  |  |  |  |  |  |  |  |  |  |  |  |  |  |  |  |  |
|  |  |  |  |  |  |  |  |  |  |  |  |  |  |  |  |  |  |  |  |  |  |  |  |  |  |  |  |  |
|  |  |  |  |  |  |  |  |  |  |  |  |  |  |  |  |  |  |  |  |  |  |  |  |  |  |  |  |  |
|  |  |  |  |  |  | Classification  10 approaches | | | | | | | |  |  |  |  |  |  |  |  |  |  |  |  |  |  |  |
|  |  |  |  |  |  |  |  |  |  |  |  |  |  |  |  |  |  |  |  |  |  |  |  |  |  |  |  |  |
|  |  |  |  |  |  |  |  |  |  |  |  |  |  |  |  |  |  |  |  |  |  |  |  |  |  |  |  |  |
|  |  |  |  |  |  |  |  |  |  |  |  |  |  |  |  |  |  |  |  |  |  |  |  |  |  |  |  |  |
|  |  |  |  |  |  |  |  |  |  |  |  |  |  |  |  |  |  |  |  |  |  |  |  |  |  |  |  |  |
|  |  |  |  |  |  | 318 classifiers | | | | | | | |  |  |  |  |  |  |  |  |  |  |  |  |  |  |  |
|  |  |  |  |  |  |  |  |  |  |  |  |  |  |  |  |  |  |  |  |  |  |  |  |  |  |  |  |  |
|  |  |  |  |  |  |  |  |  |  |  |  |  |  |  |  |  |  |  |  |  |  |  |  |  |  |  |  |  |
|  |  |  |  |  |  |  |  |  |  |  |  |  |  |  |  |  |  |  |  |  |  |  |  |  |  |  |  |  |
|  |  |  |  |  |  |  |  |  |  |  |  |  |  |  |  |  |  |  |  |  |  |  |  |  |  |  |  |  |
|  |  |  |  |  |  | AUC ≧ 0.7 and Sensitivity ≧ 0.5  2 classifiers | | | | | | | |  |  |  |  |  |  |  |  |  |  |  |  |  |  |  |
|  |  |  |  |  |  |  |  |  |  |  |  |  |  |  |  |  |  |  |  |  |  |  |  |  |  |  |  |  |
|  |  |  |  |  |  |  |  |  |  |  |  |  |  |  |  |  |  |  |  |  |  |  |  |  |  |  |  |  |
|  |  |  |  |  |  |  |  |  |  |  |  |  |  |  |  |  |  |  |  |  |  |  |  |  |  |  |  |  |
|  |  |  |  |  |  |  |  |  |  |  |  |  |  |  |  |  |  |  |  |  |  |  |  |  |  |  |  |  |
|  |  |  |  |  |  |  |  |  |  |  | Classification using test data.  2 classifiers | | | | | | | |  |  |  |  |  |  |  |  |  |  |
|  |  |  |  |  |  |  |  |  |  |  |  |  |  |  |  |  |  |  |  |  |  |  |  |  |  |  |  |  |
|  |  |  |  |  |  |  |  |  |  |  |  |  |  |  |  |  |  |  |  |  |  |  |  |  |  |  |  |  |
|  |  |  |  |  |  |  |  |  |  |  |  |  |  |  |  |  |  |  |  |  |  |  |  |  |  |  |  |  |
|  | AUC= area under the curve | | | | | | | | | | | | | | | | | | | | | | | | | | | |

**Machine learning methods**

We selected the variables relating to clinical and laboratory criteria that define a diagnosis for sepsis from the original database. We divided the data on severe maternal outcome (SMO) into two categories – yes or no, to represent whether or not the outcome occurred. This was then added to the dataset as the outcome of the analysis. We managed three databases that included all the criteria despite being collected at different times. Database 1 included all data collected on day -1 (immediately before infection suspicion or diagnosis); database 2 included data collected on the day the infection was suspected or diagnosed; and database 3 included data collected on day +1 (immediately after infection suspicion or diagnosis).

The following describes the process systematically followed with each of the three databases. Data were imported into WEKA software (*Waikato Environment for Knowledge Analysis*, version 3.6.0, 2014) (1), randomly ordered, and numerical attributes were normalised between 0 and 1, which helps ensure that no one variable has too much influence on the results. We kept the parameters of the two functions used with their default values. Reorganisation is important to remove potential biases in data collection or digitisation sequencing. Normalisation avoids distortions that a wide range of variables can cause during classifications. In the last step in the pre-processing stage of data, all databases were split into training (80% of data) and testing (20% of data), keeping the distribution of SMO similar in both.

The steps relating to selecting attributes and training classifiers were completed using the training dataset that included most of the data. To identify the most important factors for identification of SMO, we used two approaches of attribute selection: filter and *wrapper*. In the filter method, we used techniques called the “ranker search”, “information gain” and “gain ratio” to rank the characteristics based on their usefulness. For the *wrapper* approach, we used eight classification algorithms: logistic, J48, Random Forest, Naïve Bayes, PART, IBk3, IBk5 and Multilayer Perceptron. The search algorithm and Best First were used in all cases. Apart from proximity numbers in the IBk algorithm, no other parameter was modified. We applied ten attribute selection techniques to all three original databases to create a new database for each tested method.

After selecting the most important factors using the previous methods, we used WEKA’s *Experimenter* interface to build models (classifiers) and measure how well they can be used to identify SMO. We tested nine classification algorithms: logistic, J48, KStar, Random Forest, Naïve Bayes, PART, IBk3, IBk5 and Multilayer Perceptron. We only modified the proximity numbers from the IBk algorithm, and all other parameters were used with the default values. We performed ten tests for each algorithm using the cross-fold technique, where the number of folds was equal to 10. Based on the results obtained from the *Experimenter*, we developed a script for R to calculate the mean of the metrics of the ten tests performed in each algorithm. The predictive metrics that were assessed were number of true positives and false positives, the number of true negatives and false negatives, and from these, we calculated sensitivity, specificity, false positive rate, false negative rates, positive predictive value, negative predictive value, area under the ROC curve, odds ratios with a 95% confidence interval. Lastly, we also calculated the index of maximum accuracy (IMA).

To assess the best-trained classifiers, we defined cut-off points to identify which two would be evaluated. The cut-off points were sensitivity ≥0.5 and ROC ≥0.7. All classifiers with values above the cut-off points were used to assess the testing database (20% of the original database). This is a crucial step as it assesses the capacity of each classifier among unknown data, calculated or a factual error in classification.

References

1. Hall M, Frank E, Holmes G, Pfahringer B, Reutemann P, Witten IH. The WEKA Data Mining Software: An Update.

| **Table S5. Variables used to for the construction of all 28 scores and warning signals found in the literature search.** | | | | | | | | | | | | | | | | | | | | | | | | | |  |  |
| --- | --- | --- | --- | --- | --- | --- | --- | --- | --- | --- | --- | --- | --- | --- | --- | --- | --- | --- | --- | --- | --- | --- | --- | --- | --- | --- | --- |
| **Variable**    **Score** | **FAST-M** | **FAST-M red flag** | **IMEWS** | **MEOWS A** | **MEOWS B** | **MEOWS C** | **MEOWS D** | **MEOWS E** | **MEOWS F** | **MEWT (pregnant women)** | **NICE-RST (pregnancy)** | **NICE-RST (postpartum/ post-abortion)** | **qSOFA** | **SIRS** | **SOS** | **mSIRS** | **Modified SI** | **OmqSOFA** | **qSOFA-P** | **EMIP** | **MARSHALL SCORE** | ***CMQCC*** | ***APACHE 2*** | ***eCART*** | ***SEP-1*** | ***SAP 2*** | **CIPHER** |
| **Baseline information** | | | | | | | | | | | | | | |  |  |  |  |  |  |  |  |  |  |  |  |  |
| Corticosteroids during pregnancy |  |  |  |  |  |  |  |  |  |  |  |  |  |  |  |  |  |  |  |  |  |  |  |  |  |  |  |
| Blood |  |  |  |  |  |  |  |  |  |  |  |  |  |  |  |  |  |  |  |  |  |  |  |  |  |  |  |
| Immunosuppressors |  |  |  |  |  |  |  |  |  |  |  |  |  |  |  |  |  |  |  |  |  |  |  |  |  |  |  |
| Chemotherapy |  |  |  |  |  |  |  |  |  |  |  |  |  |  |  |  |  |  |  |  |  |  |  |  |  |  |  |
| Age |  |  |  |  |  |  |  |  |  |  |  |  |  |  |  |  |  |  |  |  |  |  |  |  |  |  |  |
| **Clinical signs at presentation** | | | | | | | | | | | | | | |  |  |  |  |  |  |  |  |  |  |  |  |  |
| Altered mental status/Glasgow coma score |  |  |  |  |  |  |  |  |  |  |  |  |  |  |  |  |  |  |  |  |  |  |  |  |  |  |  |
| Temperature |  |  |  |  |  |  |  |  |  |  |  |  |  |  |  |  |  |  |  |  |  |  |  |  |  |  |  |
| Respiratory rate |  |  |  |  |  |  |  |  |  |  |  |  |  |  |  |  |  |  |  |  |  |  |  |  |  |  |  |
| Heart rate |  |  |  |  |  |  |  |  |  |  |  |  |  |  |  |  |  |  |  |  |  |  |  |  |  |  |  |
| Systolic blood pressure |  |  |  |  |  |  |  |  |  |  |  |  |  |  |  |  |  |  |  |  |  |  |  |  |  |  |  |
| Diastolic blood pressure |  |  |  |  |  |  |  |  |  |  |  |  |  |  |  |  |  |  |  |  |  |  |  |  |  |  |  |
| Urine passing/output |  |  |  |  |  |  |  |  |  |  |  |  |  |  |  |  |  |  |  |  |  |  |  |  |  |  |  |
| Pulse oximetry |  |  |  |  |  |  |  |  |  |  |  |  |  |  |  |  |  |  |  |  |  |  |  |  |  |  |  |
| *Mean Arterial pressure* |  |  |  |  |  |  |  |  |  |  |  |  |  |  |  |  |  |  |  |  |  |  |  |  |  |  |  |
| **Hemogram** | | | | | | | | | | | | | | |  |  |  |  |  |  |  |  |  |  |  |  |  |
| WBC count |  |  |  |  |  |  |  |  |  |  |  |  |  |  |  |  |  |  |  |  |  |  |  |  |  |  |  |
| *Hematocrit (%)* |  |  |  |  |  |  |  |  |  |  |  |  |  |  |  |  |  |  |  |  |  |  |  |  |  |  |  |
| *Platelet count* |  |  |  |  |  |  |  |  |  |  |  |  |  |  |  |  |  |  |  |  |  |  |  |  |  |  |  |
| **Gasometry** | | | | | | | | | | | | | | |  |  |  |  |  |  |  |  |  |  |  |  |  |
| Oxygen saturation |  |  |  |  |  |  |  |  |  |  |  |  |  |  |  |  |  |  |  |  |  |  |  |  |  |  |  |
| PaCO2 |  |  |  |  |  |  |  |  |  |  |  |  |  |  |  |  |  |  |  |  |  |  |  |  |  |  |  |
| *Oxygenation FiO2* |  |  |  |  |  |  |  |  |  |  |  |  |  |  |  |  |  |  |  |  |  |  |  |  |  |  |  |
| *PaO2* |  |  |  |  |  |  |  |  |  |  |  |  |  |  |  |  |  |  |  |  |  |  |  |  |  |  |  |
| **Other labs** | | | | | | | | | | | | | | |  |  |  |  |  |  |  |  |  |  |  |  |  |
| Lactate |  |  |  |  |  |  |  |  |  |  |  |  |  |  |  |  |  |  |  |  |  |  |  |  |  |  |  |
| *Arterial pH* |  |  |  |  |  |  |  |  |  |  |  |  |  |  |  |  |  |  |  |  |  |  |  |  |  |  |  |
| *Bicarbonate* |  |  |  |  |  |  |  |  |  |  |  |  |  |  |  |  |  |  |  |  |  |  |  |  |  |  |  |
| *Bilirubin* |  |  |  |  |  |  |  |  |  |  |  |  |  |  |  |  |  |  |  |  |  |  |  |  |  |  |  |
| *Serum creatinine* |  |  |  |  |  |  |  |  |  |  |  |  |  |  |  |  |  |  |  |  |  |  |  |  |  |  |  |
| *BUN* |  |  |  |  |  |  |  |  |  |  |  |  |  |  |  |  |  |  |  |  |  |  |  |  |  |  |  |
| *Serum glucose* |  |  |  |  |  |  |  |  |  |  |  |  |  |  |  |  |  |  |  |  |  |  |  |  |  |  |  |
| *aPTT* |  |  |  |  |  |  |  |  |  |  |  |  |  |  |  |  |  |  |  |  |  |  |  |  |  |  |  |
| *Electrolytes* |  |  |  |  |  |  |  |  |  |  |  |  |  |  |  |  |  |  |  |  |  |  |  |  |  |  |  |
| *Liver function* |  |  |  |  |  |  |  |  |  |  |  |  |  |  |  |  |  |  |  |  |  |  |  |  |  |  |  |
| *Coagulation status (PT, INR, PTT)* |  |  |  |  |  |  |  |  |  |  |  |  |  |  |  |  |  |  |  |  |  |  |  |  |  |  |  |
| **Other clinical signs** | | | | | | | | | | | | | | |  |  |  |  |  |  |  |  |  |  |  |  |  |
| Decreased capillarity refill or mottling |  |  |  |  |  |  |  |  |  |  |  |  |  |  |  |  |  |  |  |  |  |  |  |  |  |  |  |
| Fetal heart rate |  |  |  |  |  |  |  |  |  |  |  |  |  |  |  |  |  |  |  |  |  |  |  |  |  |  |  |
| **Primary source of infection** | | | | | | | | | | | | | | |  |  |  |  |  |  |  |  |  |  |  |  |  |
| Endometritis |  |  |  |  |  |  |  |  |  |  |  |  |  |  |  |  |  |  |  |  |  |  |  |  |  |  |  |
| Skin/soft tissue |  |  |  |  |  |  |  |  |  |  |  |  |  |  |  |  |  |  |  |  |  |  |  |  |  |  |  |
| **Complications** | | | | | | | | | | | | | | |  |  |  |  |  |  |  |  |  |  |  |  |  |
| Postpartum haemorrhage |  |  |  |  |  |  |  |  |  |  |  |  |  |  |  |  |  |  |  |  |  |  |  |  |  |  |  |
| Abortion-related haemorrhage |  |  |  |  |  |  |  |  |  |  |  |  |  |  |  |  |  |  |  |  |  |  |  |  |  |  |  |
| Prolonged rupture of membranes |  |  |  |  |  |  |  |  |  |  |  |  |  |  |  |  |  |  |  |  |  |  |  |  |  |  |  |
| Manual removal of placenta |  |  |  |  |  |  |  |  |  |  |  |  |  |  |  |  |  |  |  |  |  |  |  |  |  |  |  |
| *Mechanical ventilation/CPAP* |  |  |  |  |  |  |  |  |  |  |  |  |  |  |  |  |  |  |  |  |  |  |  |  |  |  |  |
| *ICU stays* |  |  |  |  |  |  |  |  |  |  |  |  |  |  |  |  |  |  |  |  |  |  |  |  |  |  |  |
| *Ward hours* |  |  |  |  |  |  |  |  |  |  |  |  |  |  |  |  |  |  |  |  |  |  |  |  |  |  |  |
| *Chronic diseases* |  |  |  |  |  |  |  |  |  |  |  |  |  |  |  |  |  |  |  |  |  |  |  |  |  |  |  |
| *Type of admission (scheduled, unscheduled)* |  |  |  |  |  |  |  |  |  |  |  |  |  |  |  |  |  |  |  |  |  |  |  |  |  |  |  |
| *Surgery in the past 24hrs* |  |  |  |  |  |  |  |  |  |  |  |  |  |  |  |  |  |  |  |  |  |  |  |  |  |  |  |

Scores in black were included in the analysis. Scores highlighted in red were excluded because the GLOSS database did not contain the necessary variables for their computation.FAST-M: fluids, Antibiotics, Fluids, Antibiotics, source identification and control, transfer to an appropriate level of care, and ongoing monitoring of mother and neonate; FAST-M_RF: Fluids, Antibiotics, source identification and control, transfer to a proper level of care, and ongoing monitoring of mother and neonate red flags; IMEWS: Irish Maternity Early Warning System; MEOWS A-F: Modified early obstetric warning score; MEWT: Maternal Early Warning Trigger; NICE-RST-P: National Institute for Health and Care Excellence Risk Stratification Tool for pregnancy; NICE-RST-PP/PA: National Institute for Health and Care Excellence Risk Stratification Tool for postpartum or post-abortion; qSOFA: quick Sequential Organ Failure Assessment; SIRS: Systemic inflammatory response syndrome; SOS: Sepsis in Obstetrics Score; mSIRS: maternity systemic inflammatory response syndrome; OmqSOFA: Obstetrically modified quick Sequential Organ Failure Assessment; qSOFA-P: quick Sequential Organ Failure Assessment in Pregnancy; EMIP: Early Maternal Infection Prompt; SI: Shock Index; MSI: Modified Shock Index; mSIRS

Table S6. Early warning systems and trigger thresholds for each score. Shadowed boxes mean that those variables are needed for the specific EWS.

| **Variable**    **Score** | **FAST-M** | **FAST-M red flag** | **IMEWS** | **MEOWS A** | **MEOWS B** | **MEOWS C** | **MEOWS D** | **MEOWS E** | **MEOWS F** | **MEWT (pregnant women)** | **NICE-RST (pregnancy)** | **NICE-RST (postpartum/postabortion)** | **qSOFA** | **SIRS** | **SOS** | **mSIRS** | **OmqSOFA** | **qSOFA-P** | **EMIP** | **SI** | **MSI** |
| --- | --- | --- | --- | --- | --- | --- | --- | --- | --- | --- | --- | --- | --- | --- | --- | --- | --- | --- | --- | --- | --- |
| **Baseline information** | | | | | | | | | | | | | | | | | | | |  |  |
| Steroids for preterm labour (Y/N) |  |  |  |  |  |  |  |  |  |  | Yes | Yes |  |  |  |  |  |  |  |  |  |
| Blood products/transfusion (Y/N) |  |  |  |  |  |  |  |  |  |  | Yes | Yes |  |  |  |  |  |  |  |  |  |
| Immunosuppressors (Y/N) |  |  |  |  |  |  |  |  |  |  | Yes | Yes |  |  |  |  |  |  |  |  |  |
| Chemotherapy (Y/N) |  |  |  |  |  |  |  |  |  |  | Yes | Yes |  |  |  |  |  |  |  |  |  |
| **Clinical signs at presentation** | | | | | | | | | | | | | | | | | | | |  |  |
| Altered mental status/Glasgow coma score | Altered | Altered | Altered | Altered | Altered | Altered | Altered | Altered | Altered | Altered | Altered | Altered | Altered | Altered |  | Altered | Altered | Altered |  |  |  |
| Temperature | <36  or  >38 | <35 | ≧37.4 or ≦36 | <36  or  ≧38 | <36  or  >37.9 | <36  or  >37.9 | <36  or  ≧38 | <36  or  ≧38 | ≦35  or  ≧38 | <35.9  or  >37.9 |  | <36 |  | <36  Or  >38.3 | ≧38.5 | <36  or  >38 |  |  | ≧37.5 |  |  |
| Respiratory rate (bpm) | 21-24 | <10  or  ≧25 | >19  or  <11 | ≧21  or  <11 | ≧21  or  ≦10 | >20  or  ≦10 | <15  or  ≧20 | ≦10  or  ≧20 | <10  or  ≧18 | <12 or >24 | ≧25 | ≧21-24 | ≧22 | >20 | ≧25 | >20 | ≧25 | ≧35 | ≧20 |  |  |
| Heart rate (bpm) | 100-119 | ≧120 | <60  or  >99 | >110  or  <60 | <50  or  ≧160 | ≦50  Or  ≧100 | <75  or  ≧105 | ≦50  or  ≧110 | <60  or ≧111 | <50  or  >110 | ≧130 | ≧100-130 |  | >90 | ≧120 | >100 |  |  | ≧100 |  |  |
| Systolic blood pressure (mmHg) | 90-99 | <90 | <100  or  >139 | ≧150  or  <90 | <90 to >160 | <100  or  ≧150 | <90  Or  ≧140 | ≦100  or  ≧150 | <90  or ≧140 | <80  or  >155 | <90 or > 40 from norm | 90-100 | ≦100 |  |  | <90 | ≦90 | ≦85 | ≦100 |  |  |
| Diastolic blood pressure (mmHg) | 90 -100 | > 110 | <50 | >89 | >100 | ≧90 |  | <40 | ≧90 | <45 |  |  |  |  |  |  |  |  |  |  |  |
| Urine passing/output (Y/N or ml/24h) | None in last 12 -18 hrs | None in > 18 hrs |  |  |  |  |  |  |  |  | None in > 18 hrs | None in 12-18hrs |  |  |  |  |  |  |  |  |  |
| Pulse oximetry (%) | <95 | <95 | <95 | <90 | <95 | <95 | <95 | <95 | <95 | ≦93 | <92 | <92 |  |  | >91 |  |  |  |  |  |  |
| **Hemogram** | | | | | | | | | | | | | | | | | | | |  |  |
| WBC count (x10^9^/L) |  |  |  |  |  |  |  |  |  |  |  |  |  | <4  Or  >12 | >17 | <4  Or  >16 |  |  |  |  |  |
| **Gasometry** | | | | | | | | | | | | | | | | | | | |  |  |
| Oxygen supplementation |  |  |  |  |  |  |  |  |  |  | Yes | Yes |  |  |  |  |  |  |  |  |  |
| PaCO2 |  |  |  |  |  |  |  |  |  |  |  |  |  | <32 mmHg |  | <32 mmHg |  |  |  |  |  |
| **Other labs** | | | | | | | | | | | | | | | | | | | |  |  |
| Lactate (mmol/l) |  |  |  |  |  |  |  |  |  |  | ≧2 | ≧2 |  |  | ≧4 |  |  |  |  |  |  |
| Glucose (mmol/L) |  |  |  |  |  |  |  |  |  |  |  |  |  | >7.7 |  |  |  |  |  |  |  |
| **Other clinical signs** | | | | | | | | | | | | | | | | | | | |  |  |
| Decreased capillarity refill or mottling |  |  |  |  |  |  |  |  |  |  | Yes | Yes |  |  |  |  |  |  |  |  |  |
| Fetal heart rate >160bpm (Y/N) |  |  |  |  |  |  |  |  |  | Yes |  |  |  |  |  |  |  |  |  |  |  |
| **Primary source of infection** | | | | | | | | | | | | | | | | | | | |  |  |
| Endometritis |  |  |  |  |  |  |  |  |  |  |  | Yes |  |  |  |  |  |  |  |  |  |
| Skin/soft tissue |  |  |  |  |  |  |  |  |  |  | Yes | Yes |  |  |  |  |  |  |  |  |  |
| **Complications** | | | | | | | | | | | | | | | | | | | |  |  |
| Postpartum haemorrhage |  |  |  |  |  |  |  |  |  |  |  | Yes |  |  |  |  |  |  |  |  |  |
| Abortion-related haemorrhage |  |  |  |  |  |  |  |  |  |  |  | Yes |  |  |  |  |  |  |  |  |  |
| Prolonged rupture of membranes |  |  |  |  |  |  |  |  |  |  | Yes |  |  |  |  |  |  |  |  |  |  |
| Manual removal of placenta |  |  |  |  |  |  |  |  |  |  |  | Yes |  |  |  |  |  |  |  |  |  |
| Trigger threshold | ≧ 2 | ≧ 1 red flag | ≧ 2 yellow or >1 red | ≧ 2 amber or >1 red | ≧ 2 amber or >1 red | ≧ 2 amber or >1 red | Score 4-5 = medium risk; Score ≧6 = high risk | Score of 4-5 medium risk; Score ≧6 = high risk | ≧ 2 criteria met | ≧ 2 criteria met | Meet any criteria | Meet any criteria | ≧ 2 criteria met | ≧ 2 criteria met | Sum of score > 5 | ≧ 2 criteria met | ≧ 2 criteria met | ≧ 2 criteria met | ≧ 2 criteria met | ≧ 0.9 | ≧ 0.85 |
| Shadowed boxes mean that those variables are needed for the specific EWS. FAST-M: fluids, Antibiotics, Fluids, Antibiotics, source identification and control, transfer to an appropriate level of care, and ongoing monitoring of mother and neonate; FAST-M_RF: Fluids, Antibiotics, source identification and control, transfer to a proper level of care, and ongoing monitoring of mother and neonate red flags; IMEWS: Irish Maternity Early Warning System; MEOWS A-F: Modified early obstetric warning score; MEWT: Maternal Early Warning Trigger; NICE-RST-P: National Institute for Health and Care Excellence Risk Stratification Tool for pregnancy; NICE-RST-PP/PA: National Institute for Health and Care Excellence Risk Stratification Tool for postpartum or post-abortion; qSOFA: quick Sequential Organ Failure Assessment; SIRS: Systemic inflammatory response syndrome; SOS: Sepsis in Obstetrics Score; mSIRS: maternity systemic inflammatory response syndrome; OmqSOFA: Obstetrically modified quick Sequential Organ Failure Assessment; qSOFA-P: quick Sequential Organ Failure Assessment in Pregnancy; EMIP: Early Maternal Infection Prompt; SI: Shock Index; MSI: Modified Shock Index. | | | | | | | | | | | | | | | | | | | |  |  |

Table S7. Characteristic of the study population (N = 2,560)

| Characteristics | Women | |
| --- | --- | --- |
|  | N = 2560 | (%) |
| Age (years) |  |  |
| <20 | 353 | (13.8%) |
| 20-35 | 1890 | (74.0%) |
| >35 | 312 | (12.2%) |
|  |  |  |
| Living with partner/spouse | 2080 | (81.3%) |
|  |  |  |
| Schooling (years) |  |  |
| <5 years | 174 | (9.9%) |
| 5-8 years | 327 | (18.6%) |
| 9-11 years | 643 | (36.6%) |
| >11 years | 611 | (34.8%) |
|  |  |  |
| Number of previous births |  |  |
| 0 | 1103 | (43.8%) |
| 1-2 | 970 | (38.5%) |
| >2 | 448 | (17.7%) |
|  |  |  |
| Location at the time of infection suspected or confirmed |  |  |
| Arriving from home | 1288 | (50.5%) |
| Transferred from another facility | 378 | (14.8%) |
| Already hospitalised | 886 | (34.7%) |
|  |  |  |
| Pregnancy status at the time of infection suspected/diagnosed |  | |
| Pregnant, not in labour | 889 | (34.7%) |
| Pregnant, in labour | 309 | (12.1%) |
| Postpartum | 1100 | (43.0%) |
| Post-abortion ^a^ | 261 | (10.2%) |
|  |  |  |
| Other complications or diseases | |  |
| Anaemia during pregnancy | 799 | (31.2%) |
| Pregnancy-related hypertension | 296 | (11.6%) |
| Pre-existing medical condition | 169 | (6.6%) |
| Postpartum haemorrhage | 171 | (6.7%) |
| Obstructed labour or dystocia | 117 | (4.6%) |
| Abortion-related haemorrhage | 131 | (5.12%) |
|  |  |  |
| Pregnancy outcome ^b^ |  |  |
| Still pregnant | 618 | (24.1%) |
| Abortion ^a^ | 307 | (12.0%) |
| Stillbirth | 132 | (5.2%) |
| Livebirth | 1491 | (58.2%) |
|  |  |  |
| Final mode of birth ^b, c^ |  |  |
| Vaginal birth | 802 | (44.2%) |
| Caesarean section | 1012 | (55.8%) |
|  |  |  |
| Maternal status at end of follow-up |  |  |
| Discharged alive | 2775 | (97.5%) |
| Transferred | 45 | (1.6%) |
| Dead | 26 | (0.9%) |

^a^ women with abortion, ectopic (n=36) and molar (n=7) pregnancy;

^b^ includes data on multiple pregnancies.

^c^ women with childbirth (stillbirth or live birth).

Table S8. Percentages of missing values for clinical signs and symptoms on enrolment into the GLOSS.

| Parameter | Missingness Overall (%) | Missingness Day -1 (%) | Missingness Day 0 (%) | Missingness  Day +1 (%) |
| --- | --- | --- | --- | --- |
| Altered mental status | 6.5 | 45.5 | 7.2 | 13.5 |
| Glasgow Coma Score | 50.4 | 79.6 | 21.9 | 55.95 |
| Temperature (highest) | 50.4 | 57.9 | 11.3 | 21.3 |
| Temperature (Lowest) | 14.0 | 61.9% | 17.5% | 25.3% |
| Respiratory Rate (highest) | 25.1 | 66.2% | 29.4% | 35.2 |
| Respiratory Rate (lowest) | 27.6 | 67.8% | 31.5% | 37.4 |
| Systolic Blood pressure (Highest) | 8.9 | 58.2% | 11.3% | 21.4 |
| Systolic Blood pressure (lowest) | 11.9 | 60.2% | 15.2% | 24.7 |
| Diastolic Blood pressure (lowest) | 6.6 | 57.1% | 8.8% | 19.2 |
| Urine Passing | 13.0 | 53.3% | 14.5% | 19.8 |
| Urine output | 67.8 | 84.2% | 70.8% | 73.2 |
| Pulse Oximetry | 66.8 | 87.4% | 69.3% | 75.0 |
| WBC Count | 18.9 | 71.2% | 34.9% | 65.3 |
| PaC02 | 92.7 | 98.0% | 94.3% | 96.3 |
| Hemogram | 24.7 | 74.8 | 38.4 | 67.3 |
| Lactate | 93.4 | 98.2 | 95.1 | 96.9 |
| Decreased capillary refill or mottling | 25.6 | 60.8 | 26.3 | 29.1 |
| Fetal Heart Rate | 22.2 | 64.7 | 24.2 | 29.6 |
| Blood transfusion | 56.2 |  |  |  |
| Immunosuppressors | 10.2 |  |  |  |
| Chemotherapy | 9.2 |  |  |  |
| Endometritis | 18.4 |  |  |  |
| Postpartum Haemorrhage | 10.5 |  |  |  |
| Abortion-related haemorrhage | 8.5 |  |  |  |
| Manual removal of the placenta | 30.0 |  |  |  |

Shading indicates red >75% missing; orange >50% but ≤75% missing; yellow >25% but ≤50% missing; and green ≤25% missing. Cells in white mean there is no data available.

| Table S9a. Diagnostic accuracy for sepsis-related severe maternal outcomes (near-miss or death) for women from High and Upper-Middle-Income Countries (N=105). | | | | | | | | |
| --- | --- | --- | --- | --- | --- | --- | --- | --- |
| Early warning system | Sensitivity | Specificity | Positive Likelihood Ratio (95%CI) | Negative Likelihood Ratio (95% CI) | Positive Predictive Value  (95% CI) | Negative Predictive Value  (95% CI) | Diagnostic Odds Ratio  (95% CI) | J-statistic Youden’s index (95% CI) |
| *Obstetric scores – not sepsis specific* | | | | | | | | |
| FAST-M | 46.67 | 72.57 | 1.70  (1.14 -2.55) | 0.73  (0.57-0.96) | 0.47  (0.34-0.61) | 0.72  (0.63-0.8) | 2.31  (1.2 -4.45) | 0.19  (-0.03-0.41) |
| FAST - red flag | 40 | 82.61 | 2.3  (1.47-3.60) | 0.73  (0.6-0.88) | 0.59  (0.45-0.71) | 0.69  (0.61-0.76) | 3.17  (1.71-5.88) | 0.23  (0.05-0.4) |
| IMEWS | 75.27 | 34.64 | 1.15  (0.98-1.36) | 0.71  (0.47-1.08) | 0.41  (0.34-0.49) | 0.7  (0.58-0.8) | 1.61  (0.91-2.87) | 0.1  (-0.08-0.26) |
| MEOWS A | 73.12 | 46.41 | 1.36  (1.13-1.65) | 0.58  (0.4-0.84) | 0.45  (0.37-0.54) | 0.74  (0.64-0.82) | 2.36  (1.35-4.11) | 0.19  (0.01-0.36) |
| MEOWS B | 68.81 | 54.25 | 1.50  (1.21-1.87) | 0.57  (0.41-0.80) | 0.48  (0.39-0.57) | 0.74  (0.65-0.82) | 2.62  (1.52-4.5) | 0.23  (0.04-0.4) |
| MEOWS C | 69.89 | 52.94 | 1.49  (1.2-1.84) | 0.57  (0.4-0.8) | 0.47  (0.39-0.56) | 0.74  (0.65-0.82) | 2.61  (1.51-4.5) | 0.23  (0.04 -0.4) |
| MEOWS D | 48.94 | 91.94 | 2.71  (1.83-4.02) | 0.62  (0.5-0.77) | 0.62  (0.5-0.73) | 0.73  (0.65-0.79) | 4.35  (2.45-7.73) | 0.31  (0.12-0.47) |
| MEOWS E | 49.46 | 81.70 | 2.70  (1.82-4.0) | 0.62  (0.5-0.77) | 0.62  (0.5-0.73) | 0.73  (0.65-0.79) | 4.37  (2.45-7.81) | 0.31  (0.14-0.48) |
| MEOWS F | 67.39 | 59.87 | 1.68  (1.32-2.14) | 0.55  (0.39-0.75) | 0.5  (0.41-0.6) | 0.75  (0.67-0.83) | 3.08  (1.79-5.31) | 0.27  (0.08-0.45) |
| MEWT (pregnant) | 25.58 | 86.96 | 1.96  (0.79-4.84) | 0.86  (0.7-1.05) | 0.65  (0.38-0.86) | 0.56  (0.43-0.67) | 2.29  (0.76-6.87) | 0.13  (-0.13-0.36) |
| SOS | 12.50 | 99.35 | 19.38  (2.56-146.65) | 0.88  (0.82-0.95) | 0.92  (0.64-1.0) | 0.65  (0.58-0.71) | 22  (2.81-172.14) | 0.12  (0.03-0.21) |
| *Obstetric scores – sepsis specific* | | | | | | | | |
| UKST-postpartum/postabortion | 95.65 | 13.33 | 1.10  (0.96-1.27) | 0.33  (0.04-2.55) | 0.36  (0.24-0.49) | 0.86  (0.42-1.0) | 3.38  (0.38-29.95) | 0.08  (-0.17-0.27) |
| UKST-pregnant | 65.71 | 31.58 | 0.96  (0.7-1.33) | 1.09  (0.56-2.09) | 0.47  (0.33-0.62) | 0.5  (0.29-0.71) | 0.88  (0.33-2.35) | -0.03  (-0.34-0.3) |
| mSIRS | 84.44 | 26.92 | 1.16  (0.94-1.42) | 0.58  (0.26-1.31) | 0.5  (0.38-0.62) | 0.67  (0.43-0.85) | 2  (0.73-5.51) | 0.11  (-0.14-0.35) |
| OmqSOFA | 35.19 | 91.38 | 4.08  (1.64-10.17) | 0.71  (0.57-0.88) | 0.79  (0.58-0.93) | 0.6  (0.49-0.71) | 5.75  (1.97-16.84) | 0.27  (0.04-0.47) |
| qSOFA-P | 20.83 | 100 | *INF | 0.79  (0.68-0.92) | 1.0  (0.69-1) | 0.59  (0.48-0.69) | *INF  (NAN-INF) | 0.21  (0.04-0.35) |
| EMIP | 84.62 | 20.59 | 1.07  (0.9-1.26) | 0.75  (0.34-1.65) | 0.45  (0.35-0.55) | 0.64  (0.41-0.83) | 1.43  (0.55-3.71) | 0.05  (-0.16-0.25) |
| *Scores -not obstetric or sepsis specific* | | | | | | | | |
| qSOFA^¶^ | 33.70 | 88 | 2.81  (1.67-4.72) | 0.75  (0.64-0.88) | 0.63  (0.48-0.77) | 0.68  (0.61-0.75) | 3.73  (1.94-7.18) | 0.22  (0.06-0.37) |
| SIRS^¶^ | 58.43 | 68.15 | 1.83  (1.36-2.48) | 0.61  (0.46-0.8) | 0.55  (0.44-0.65) | 0.71  (0.63-0.79) | 3.01  (1.72-5.24) | 0.27  (0.07-0.45) |
| SI | 59.3 | 62.6 | 1.59  (1.05–2.39) | 0.65  (0.4–1.05) | 0.32  (0.2–0.47) | 0.84  (0.73–0.92) | 2.44  (1.01–5.86) | 0.22  (-0.09–0.50) |
| MSI | 38.0 | 81.2 | 0.76  (0.59–0.98) | 2.03  (0.72–5.69) | 0.97  (0.91–0.99) | 0.08  (0.04–0.13) | 2.66  (0.74–9.57) | 0.19  (-0.14–0.3) |

^¶^ For women enrolled in the study during their stay in the intensive care unit (ICU)

*INF = Impossible to calculate, for the sensitivity or 1-specificity values correspond to 100% of the sample with or without sepsis

| Table S9b. Diagnostic accuracy for sepsis-related severe maternal outcomes (near-miss or death) for women from Low and Lower-Middle-Income countries (N=276). | | | | | | | | | | | | | |
| --- | --- | --- | --- | --- | --- | --- | --- | --- | --- | --- | --- | --- | --- |
| Early warning system | | Sensitivity | Specificity | Positive Likelihood Ratio (95%CI) | | Negative Likelihood Ratio (95% CI) | | Positive Predictive Value  (95% CI) | Negative Predictive Value  (95% CI) | Diagnostic Odds Ratio  (95% CI) | | | J-statistic Youden’s index (95% CI) |
| *Obstetric scores – not sepsis specific* | | | | | | | | | | | | | |
| FAST-M | 58.10 | | 71.60 | 2.05  (1.53-2.73) | 0.59  (0.46-0.75) | | 0.56  (0.46-0.65) | | 0.73  (0.66-0.8) | | 3.49  (2.09-5.83) | 0.3  (0.12-0.46) | |
| FAST - red flag | 47.92 | | 81.98 | 2.67  (1.93-3.67) | 0.64  (0.54-0.74) | | 0.67  (0.58-0.75) | | 0.67  (0.61-0.73) | | 4.19  (2.65-6.61) | 0.3  (0.16-0.43) | |
| IMEWS | 85.24 | | 33.12 | 1.27  (1.16-1.4) | 0.53  (0.31-0.64) | | 0.46  (0.41-0.52) | | 0.77  (0.69-0.84) | | 2.86  (1.82-4.48) | 0.18  (0.08-0.28) | |
| MEOWS A | 72.73 | | 52.61 | 1.54  (1.33-1.77) | 0.52  (0.41-0.66) | | 0.51  (0.45-0.57) | | 0.74  (0.67-0.8) | | 2.96  (2.03-4.32) | 0.25  (0.13-0.37) | |
| MEOWS B | 76.19 | | 49.68 | 1.51  (1.32-1.73) | 0.48  (0.37-0.63) | | 0.51  (0.45-0.56) | | 0.75  (0.69-0.81) | | 3.15  (2.69-7.25) | 0.26  (0.14-0.37) | |
| MEOWS C | 77.14 | | 48.05 | 1.49  (1.3-1.69) | 0.48  (0.36-0.63) | | 0.5  (0.45-0.56) | | 0.76  (0.69-0.81) | | 3.12  (2.11-4.62) | 0.25  (0.13-0.36) | |
| MEOWS D | 50.23 | | 82.47 | 2.87  (2.17-3.78) | 0.60  (0.52-0.7) | | 0.67  (0.59-0.74) | | 0.7  (0.65-0.75) | | 4.75  (3.19-7.06) | 0.33  (0.21-0.43) | |
| MEOWS E | 49.52 | | 80.52 | 2.54  (1.95-3.31) | 0.63  (0.54-0.72) | | 0.63  (0.56-0.71) | | 0.7  (0.65-0.75) | | 4.06  (2.74-5.99) | 0.3  (0.18-0.41) | |
| MEOWS F | 64.29 | | 66.12 | 1.9  (1.58-2.29) | 0.54  (0.44-0.66) | | 0.56  (0.5-0.63) | | 0.73  (0.67-0.78) | | 3.51  (2.43-5.08) | 0.3  (0.18-0.42) | |
| MEWT (pregnant) | 35.42 | | 90.38 | 3.68  (1.47-9.21) | 0.71  (0.57-0.9) | | 0.77  (0.55-0.93) | | 0.6  (0.49-0.71) | | 5.15  (1.72-15.41) | 0.26  (0.01-0.47) | |
| SOS | 16.89 | | 97.82 | 7.75  (3.52-17.06) | 0.85  (0.8-0.9) | | 0.84  (0.7-0.93) | | 0.63  (0.59-0.68) | | 9.12  (3.98-20.87) | 0.15  (0.08-0.22) | |
| *Obstetric scores – sepsis specific* | | | | | | | | | | | | | |
| UKST-postpartum | 98.39 | | 11.11 | 1.11  (1.03-1.19) | 0.15  (0.02-1.1) | | 0.41  (0.33-0.49) | | 0.92  (0.62-1.0) | | 7.63  (0.1-60.61) | 0.09  (-0.03-0.19) | |
| UKST-pregnant | 68.97 | | 43.33 | 1.22  (0.82-1.81) | 0.72  (0.36-1.41) | | 0.54  (0.37-0.71) | | 0.59  (0.36-0.79) | | 1.7  (0.58-4.94) | 0.12  (-0.25-0.47) | |
| mSIRS | 91.76 | | 27.78 | 1.27  (1.09-1.49) | 0.30  (0.13-0.66) | | 0.6  (0.51-0.68) | | 0.74  (0.54-0.89) | | 4.29  (1.69-10.86) | 0.2  (0.02-0.36) | |
| OmqSOFA | 39.29 | | 89.90 | 3.89  (2.07-7.31) | 0.68  (0.57-0.79) | | 0.81  (0.69-0.91) | | 0.57  (0.49-0.65) | | 5.76  (2.7-12.26) | 0.29  (0.12-0.44) | |
| qSOFA-P | 21.88 | | 100 | *INF | 0.78  (0.7-0.87) | | 1.0  (0.84-1.0) | | 0.54  (0.46-0.62) | | *INF | 0.22  (0.1-0.31) | |
| EMIP | 89.60 | | 12.71 | 1.03  (0.94-1.12) | 0.82  (0.41-1.65) | | 0.52  (0.45-0.59) | | 0.54  (0.34-0.72) | | 1.25  (0.57-2.77) | 0.02  (-0.1-0.14) | |
| *Sepsis scores -not obstetric specific* | | | | | | | | | | | | | |
| qSOFA^¶^ | 36.52 | | 80.23 | 1.85  (1.35-2.52) | 0.79  (0.7-0.9) | | 0.56  (0.46-0.65) | | 0.65  (0.6-0.7) | | 2.33  (1.52-3.59) | 0.17  (0.04-0.29) | |
| SIRS^¶^ | 65.27 | | 63.23 | 1.78  (1.45-2.18) | 0.55  (0.44-0.69) | | 0.57  (0.5-0.64) | | 0.71  (0.64-0.77) | | 3.23  (2.13-4.19) | 0.28  (0.14-0.42) | |
| SI | 72.9 | | 30.7 | 1.05  (0.88–1.25) | 0.88  (0.58–1.35) | | 0.53  (0.44–0.61) | | 0.52  (0.38–0.65) | | 1.19  (0.65–2.17) | 0.04  (-0.15–0.22) | |
| MSI | 41.1 | | 59.3 | 0.99  (0.72–1.37) | 0.05  (0.03–0.08) | | 0.95  (0.91–0.98) | | 0.05  (0.03–0.08) | | 1.02  (0.46-2.23) | -0.004  (-0.24–0.23) | |

^¶^ For women enrolled in the study during their stay in the intensive care unit (ICU)

*INF = Impossible to calculate, for the sensitivity or 1-specificity values correspond to 100% of the sample with or without sepsis

S10: Machine learning results and model databases

Table S10a. Select models with the best accuracy in the experiment.

| Model | Classification algorithm | TP | FN | FP | TN | Total  prediction | Sensitivity | Specificity | FPR | FNR | PLR | NLR | AUROC | Odds | IMA |
| --- | --- | --- | --- | --- | --- | --- | --- | --- | --- | --- | --- | --- | --- | --- | --- |
| Model3_train_Ranker_GainRatioAttributeEval_ | weka.classifiers.bayes.NaiveBayes | 154.7 | 187.3 | 119.4 | 2102.6 | 2564 | 0.50 | 1.00 | 0.05 | 0.55 | 8.42 | 0.58 | 0.76 | 14.61  (11.04 – 19.41) | 0.14 |
| Model3_train_BestFirst_WrapperSubsetEval_MultilayerPerceptron | weka.classifiers.bayes.NaiveBayes | 171.9 | 170.1 | 131.4 | 2090.6 | 2564 | 0.42 | 0.99 | 0.06 | 0.50 | 8.50 | 0.53 | 0.80 | 16.11  (12.24 – 21.28) | 0.16 |
| Model3_train_b_Ranker_InfoGainAttributeEval_ | weka.classifiers.bayes.NaiveBayes | 143.2 | 198.8 | 73.7 | 2148.3 | 2564 | 0.40 | 0.98 | 0.03 | 0.58 | 12.62 | 0.60 | 0.75 | 20.77  (15.20 – 28.62) | 0.15 |
| Model2_train_BestFirst_WrapperSubsetEval_J48 | weka.classifiers.functions.Logistic -R 1.0E-8 -M -1 -num-decimal-places 4 | 129.7 | 212.3 | 43.5 | 2178.5 | 2564 | 0.27 | 0.97 | 0.02 | 0.62 | 19.37 | 0.63 | 0.85 | 30.20  (21.02 – 44.19) | 0.17 |
| Model2_train_BestFirst_WrapperSubsetEval_NaiveBayes | weka.classifiers.functions.Logistic -R 1.0E-8 -M -1 -num-decimal-places 4 | 136.7 | 205.3 | 53.6 | 2168.4 | 2564 | 0.34 | 0.98 | 0.02 | 0.60 | 16.57 | 0.62 | 0.80 | 26.71  (19.00 – 38.08) | 0.16 |

TP – true positive; FN – false negative; FP- false positive; TN -true negative; FPR – false positive rate. FNR – false negative rate; PLR – positive likelihood ratio; MLR- negative likelihood ratio; AUROC – Area under ROC); IMA – index of maximum accuracy.

Figure S10a. Model 2 results

Figure S10b: Model 3 results

| Model 2: train_BestFirst_WrapperSubsetEval_J48 model and database | Model 2:  train_BestFirst_WrapperSubsetEval_NaiveBayes model and database | Model 3: train_b_Ranker_InfoGainAttributeEval_ model and database | Model 3: train_BestFirst_WrapperSubsetEval_PART model and database | Model 3: train_Ranker_GainRatioAttributeEval_ model and database |
| --- | --- | --- | --- | --- |
| Day 0   - Lowest temperature °C - Highest respiratory rate (breaths per minute) - Lowest respiratory rate (breaths per minute) - Highest SBP - mmHg - Lowest DBP - mmHg - Urine passing in 24h - PaCO2 – mmHg (lowest value) - Bilirubin - mg/dL (highest value) - Glucose - mg/dL (highest value) - Procalcitonin test μg/L (highest value) - Ileus (absent of bowel sounds) - Fetal heart rate > 160 - bpm - Vasopressin (highest dosage/number) - Supplemental Oxygen (highest dosage/number) - Looks unwell - Women with SMO according to if they have Maternal Near Miss or Maternal Death | Day 0   - Altered mental status - Lowest respiratory rate (breaths per minute) - Highest heart rate (beats per minute) - Highest SBP - mmHg - Lowest DBP - mmHg - Urine output (ml/24h) - Pulse-oxymetry (%) (lowest O2 saturation) - O2 supplementation at time of pulse-oxymetry - Haemoglobin g/dL (lowest value) - Hematocrit % (lowest value) - % of immature neutrophils (bands) (highest value) - Platelet count - x103 ml (lowest value) - PaCO2 - mmHg - Gasometry (lowest value) - FiO2 at the time of gasometry - Gasometry (lowest value) - aPTT (activated partial thromboplastin time) - seconds (highest value) - Procalcitonin test - μg/L (highest value) - Decreased capillarity refill or mottling - Dopamine (highest dosage/number) - Epinephrine or Norepinephrine (highest dosage/number) - Dobutamine (highest dosage/number) - Supplemental Oxygen (highest dosage/number) - Received fluids in the event of SBP < 100mmHg - Heart rate 40-49 bmp - Systolic BP 90-100 - Temperature 35.1 - 35.9°C - Temperature > 38°C - Looks unwell - Women with SMO according to if they have Maternal Near Miss or Maternal Death | Day 1   - Altered mental status - Lowest Glasgow Coma Score - Highest temperature °C - Lowest temperature °C - Highest respiratory rate (breaths per minute) - Lowest respiratory rate (breaths per minute) - Highest heart rate (beats per minute) - Lowest heart rate (beats per minute) - Highest SBP - mmHg - Lowest SBP - mmHg - Lowest DBP - mmHg - Urine passing in 24h - Urine output (ml/24h) - Pulse-oximetry (%) (lowest O2 saturation) - O2 supplementation at time of pulse-oximetry - Haemoglobin g/dL (lowest value) - Haematocrit - % (lowest value) - WBC count - mm5 - % of immature neutrophils (bands) (highest value) - Platelet count - x103 ml (lowest value) - pH (lowest value) - Lowest O2 saturation (%) (lowest value) - Oxygen partial pressure (PaO2) (lowest value) - PaCO2 - mmHg - (lowest value) - Bicarbonate HCO3 - mEq/L (lowest value) - Base excess (lowest value) - FiO2 at the time of gasometry (lowest value) - Bilirubin - mg/dL (highest value) - Creatinine - mg/dL (highest value) - Urea - mg/dL (highest value) - Lactate - mg/dL (highest value) - Glucose - mg/dL (highest value) - PT (prothrombin time) - seconds (highest value) - aPTT (activated partial thromboplastin time) - seconds (highest value) - CRP (C-reactive protein) - mg/L (highest value) - Procalcitonin test - μg/L (highest value) - Erythrocyte sedimentation rate (highest value) - Decreased capillarity refill or mottling - Ileus (absent of bowel sounds) - Fetal heart rate > 160bpm - Dopamine (highest dosage/number) - Epinephrine or Norepinephrine - Dobutamine (highest dosage/number) - Vasopressin (highest dosage/number) - Supplemental Oxygen (highest dosage/number) - Received fluids in the event of SBP < 100mmHg (highest dosage/number) - Respiration rate 21-24 rpm - Heart rate 40-49 bmp - Heart rate 100-119 bmp - Systolic BP 90-101 - Temperature 35.1 - 35.9°C - Temperature >38°C - Not passed urine in last 12-18 hrs - Looks unwell - Women with SMO according to if they have Maternal Near Miss or Maternal Death | Day 1   - Altered mental status - Lowest respiratory rate (breaths per minute) - Highest heart rate (beats per minute) - Highest SBP - mmHg - Lowest SBP - mmHg - Lowest DBP - mmHg - O2 supplementation at time of pulse-oximetry - PT (prothrombin time) seconds (highest value) - Fetal heart rate > 160 bpm - Received fluids in the event of SBP < 100mmHg (highest dosage/number) - Women with SMO according to if they have Maternal Near Miss or Maternal Death | Day 1   - Altered mental status - Lowest Glasgow Coma Score - Highest temperature °C - Lowest temperature °C - Highest respiratory rate (breaths per minute) - Lowest respiratory rate (breaths per minute) - Highest heart rate (beats per minute) - Lowest heart rate (beats per minute) - Highest SBP - mmHg - Lowest SBP - mmHg - Lowest DBP - mmHg - Urine passing in 24h - Urine output (ml/24h) - Pulse-oximetry (%) (lowest O2 saturation) - O2 supplementation at time of pulse-oximetry - Haemoglobin g/dL (lowest value) - Haematocrit - % (lowest value) - WBC count - mm5 - % of immature neutrophils (bands) (highest value) - Platelet count - x103 ml (lowest value) - pH (lowest value) - Lowest O2 saturation (%) (lowest value) - Oxygen partial pressure (PaO2) (lowest value) - PaCO2 - mmHg (lowest value) - Bicarbonate HCO3 - mEq/L (lowest value) - Base excess (lowest value) - FiO2 at the time of gasometry (lowest value) - Bilirubin - mg/dL (highest value) - Creatinine - mg/dL (highest value) - Urea - mg/dL (highest value) - Lactate - mg/dL (highest value) - Glucose - mg/dL (highest value) - PT (prothrombin time) seconds (highest value) - aPTT (activated partial thromboplastin time) seconds (highest value) - CRP (C-reactive protein) mg/L (highest value) - Procalcitonin test - μg/L (highest value) - Erythrocyte sedimentation rate (highest value) - Decreased capillarity refill or mottling - Ileus (absent of bowel sounds) - Fetal heart rate > 160 - bpm - Dopamine (highest dosage/number) - Epinephrine or Norepinephrine - Dobutamine (highest dosage/number) - Vasopressin (highest dosage/number) - Supplemental Oxygen (highest dosage/number) - Received fluids in the event of SBP < 100mmHg (highest dosage/number) - Respiration rate 21-24 rpm - Heart rate 40-49 bmp - Heart rate 100-119 bmp - Systolic BP 90-101 - Temperature 35.1 - 35.9°C - Temperature >38°C - Not passed urine in last 12-18 hrs - Looks unwell - Women with SMO according to if they have Maternal Near Miss or Maternal Death |

**S11: World Health Organization Global Maternal Sepsis Study Research Group**

| **First and middle names** | **Surnames** |
| --- | --- |

| Yamikani | Chimwaza |
| --- | --- |
| Alexandra | Hunt |
| Livia | Oliveira-Ciabati |
| Laura | Bonnett |
| Edgardo | Abalos |
| Cristina | Cuesta |
| João Paulo | Souza |
| Mercedes | Bonet |
| Vanessa | Brizuela |
| David | Lissauer |

| Mohammad Iqbal | Aman |
| --- | --- |
| Bashir | Noormal |
| Marisa | Espinoza |
| Julia | Pasquale |
| Charlotte | Leroy |
| Kristien | Roelens |
| Griet | Vandenberghe |
| M. Christian Urlyss | Agossou |
| Sourou | Goufodji Keke |
| Christiane | Tshabu Aguemon |
| Patricia Soledad | Apaza Peralta |
| Víctor | Conde Altamirano |
| Rosalinda | Hernández Muñoz |
| José Guilherme | Cecatti |
| Carolina | Ribeiro do Valle |
| Vincent | Batiene |
| Kadari | Cisse |
| Henri Gautier | Ouedraogo |
| Kannitha | Cheang |
| Phirun | Lam |
| Tung | Rathavy |
| Elie | Simo |
| Pierre-Marie | Tebeu |
| Emah Irene | Yakana |
| Javier | Carvajal |
| María Fernanda | Escobar |
| Paula | Fernández |
| Lotte Berdiin | Colmorn |
| Jens | Langhoff-Roos |
| Wilson | Mereci |
| Paola | Vélez |
| Yasser | Salah Eldin |
| Alaa | Sultan |
| Alula M. | Teklu |
| Dawit | Worku |
| Richard | Adanu |
| Philip | Govule |
| Charles | Noora Lwanga |
| William Enrique | Arriaga Romero |
| María Guadalupe | Flores Aceituno |
| Carolina | Bustillo |
| Bredy | Lara |
| Vijay | Kumar |
| Vanita | Suri |
| Sonia | Trikha |
| Irene | Cetin |
| Serena | Donati |
| Carlo | Personeni |
| Guldana | Baimussanova |
| Saule | Kabylova |
| Balgyn | Sagyndykova |
| George | Gwako |
| Alfred | Osoti |
| Zahida | Qureshi |
| Raisa | Asylbasheva |
| Aigul | Boobekova |
| Damira | Seksenbaeva |
| Saad Eddine | Itani |
| Meilė | Minkauskienė |
| Diana | Ramašauskaitė |
| Owen | Chikhwaza |
| Luis | Gadama |
| Eddie | Malunga |
| Haoua | Dembele |
| Hamadoun | Sangho |
| Fanta Eliane | Zerbo |
| Filiberto | Dávila Serapio |
| Nazarea | Herrera Maldonado |
| Juan I. | Islas Castañeda |
| Tatiana | Cauaus |
| Ala | Curteanu |
| Victor | Petrov |
| Yadamsuren | Buyanjargal |
| Seded | Khishgee |
| Bat-Erdene | Lkhagvasuren |
| Amina | Essolbi |
| Rachid | Moulki |
| Zara | Jaze |
| Arlete | Mariano |
| Nafissa | Bique Osman |
| Hla Mya Thway | Einda |
| Thae Maung | Maung |
| Khaing Nwe | Tin |
| Tara | Gurung |
| Amir Babu | Shrestha |
| Sangeeta | Shrestha |
| Kitty | Bloemenkamp |
| Marcus J. | Rijken |
| Thomas | Van Den Akker |
| María Esther | Estrada |
| Néstor J. | Pavón Gómez |
| Olubukola | Adesina |
| Chris | Aimakhu |
| Bukola | Fawole |
| Rizwana | Chaudhri |
| Saima | Hamid |
| M. Adnan | Khan |
| María del Pilar | Huatuco Hernández |
| Nelly M. | Zavaleta Pimentel |
| Maria Lu | Andal |
| Zenaida Dy | Recidoro |
| Carolina Paula | Martin |
| Mihaela | Budianu |
| Lucian | Pușcașiu |
| Léopold | Diouf |
| Dembo | Guirassy |
| Philippe Marc | Moreira |
| Miroslav | Borovsky |
| Ladislav | Kovac |
| Alexandra | Kristufkova |
| Sylvia | Cebekhulu |
| Laura | Cornelissen |
| Priya | Soma-Pillay |
| Vicenç | Cararach |
| Marta | López |
| María José | Vidal Benedé |
| Hemali | Jayakody |
| Kapila | Jayaratne |
| Dhammica | Rowel |
| Wisal | Nabag |
| Sara | Omer |
| Victoria | Tsoy |
| Urunbish | Uzakova |
| Dilrabo | Yunusova |
| Thitiporn | Siriwachirachai |
| Thumwadee | Tangsiriwatthana |
| Catherine | Dunlop |
| Marian | Knight |
| Jhon | Roman |
| Gerardo | Vitureira |
| Dinh Anh | Tuan |
| Luong Ngoc | Truong |
| Nghiem Thi Xuan | Hanh |
| Mugove | Madziyire |
| Thulani | Magwali |
| Stephen | Munjanja |
| Adama | Baguiya |
| Mónica | Chamillard |
| Bukola | Fawole |
| Marian | Knight |
| Seni | Kouanda |
| Pisake | Lumbiganon |
| Ashraf | Nabhan |
| Ruta | Nadisauskiene |
| Linda | Bartlett |
| Fernando | Bellissimo-Rodrigues |
| Shevin T. | Jacob |
| Sadia | Shakoor |
| Khalid | Yunis |
| Liana | Campodónico |
| Hugo | Gamerro |
| Daniel | Giordano |
| Fernando | Althabe |
| A. Metin | Gülmezoglu |
